# Cytotoxicity, acute and sub-chronic toxicities of the fruit extract of *Tetrapleura tetraptera (Schumm. & Thonn.) Taub.* (Fabaceae)

**DOI:** 10.1186/s12906-022-03659-1

**Published:** 2022-07-04

**Authors:** Idrios N. Bonsou, Armelle T. Mbaveng, Gaëlle S. Nguenang, Godloves F. Chi, Victor Kuete, Thomas Efferth

**Affiliations:** 1 Department of Biochemistry, Faculty of Science, University of Dschang, Dschang, Cameroon; 2 Department of Pharmaceutical Biology, Institute of Pharmaceutical and Biomedical Sciences, Johannes Gutenberg University, Staudinger Weg 5, 55128 Mainz, Germany; 3 Department of Chemistry, Faculty of Science, University of Buea, Buea, Cameroon

**Keywords:** Cytotoxicity, Fabaceae, Spice, *Tetrapleura tetraptera*, Toxicity

## Abstract

**Background:**

*Tetrapleura tetraptera* is a medicinal spice traditionally used to treat cancer, diabetes, and several other ailments. This study analyzed the cytotoxicity of the dichloromethane methanol extract of *T. tetraptera* fruits (TTF) and its constituents. The toxicity profile of the TTF extract was also evaluated in rats.

**Methods:**

The Cytotoxicity of this extract was evaluated using the resazurin reduction assay (RRA). Acute and sub-chronic toxicity studies were performed according to the protocol described by the Organisation for Economic Cooperation, and Development (OECD). Hematological, serum, and urine biochemical parameters, as well as histological sections of the liver and kidney, were also evaluated based on standard methods.

**Results:**

The TTF extract, compound 5, and the reference drug doxorubicin were active in all 9 tested cancer cell lines. The recorded IC_50_ ranged from 18.32 μM (against B16-F1 murine melanoma cells) to 36.18 μM (against SKMel-505 BRAF wildtype melanoma cells) for TTF, from 10.02 μM (towards MaMel-80a BRAF-V600E homozygous mutant melanoma cells) to 31.73 μM (against SKMel-28 BRAF-V600E homozygous mutant melanoma cells) for compound 5, and from 0.22 μM (against B16-F1 cells) to 9.39 μM (against SKMel-505 cells) for doxorubicin. The study of acute toxicity test showed that the lethal dose (LD_50_) of this extract was greater than 5000 mg/kg body weight. In the sub-chronic toxicity studies, variations were observed in some biochemical parameters, especially at higher doses.

**Conclusion:**

TTF and its most active compound (5) are found to be potential cytotoxic agents, meanwhile, TTF was safe when given a single oral dose of 5000 mg/kg. However, caution is necessary in case of prolonged oral administration due to potential alterations of renal function at high doses (> 1000 mg/kg).

**Supplementary Information:**

The online version contains supplementary material available at 10.1186/s12906-022-03659-1.

## Background

Cancer remains a global health challenge with about 19.3 million new cases and 10 million deaths recorded in 2020 [1]. In many countries, cancer's rising prominence as a leading cause of death partly reflects marked declines in mortality rates of stroke and coronary heart disease compared to cancer, [1]. However, the toxicity of anticancer drugs as well as the chemoresistance constitutes major challenges in cancer chemotherapy [2–4]. Several concepts are therefore being explored with medicinal food plants as one of the most promising ones [5–7]. Food plants commonly used in Cameroon are endowed with very good antibacterial, anticancer, antidiabetic, antiparasitic, and antiviral activities due to their richness in secondary metabolites [8–16].

*Tetrapleura tetraptera* is a widely distributed plant in West Africa. It is commonly known as *Prekese* and *Aridan* in Ghana and Nigeria, respectively, where it is highly valued for its use in traditional medicine and its nutritional properties [17]. It is a perennial plant belonging to the *Fabaceae* family, also found in the West Cameroon region, where it is traditionally used as a plant of choice in the treatment of obesity [18]. *T. tetraptera* contains several secondary metabolites belonging to different classes: flavonoids, alkaloids, phenolic compounds, tannins, and saponins [19]. The ethanolic extract of *T. tetraptera* fruits (TTF) inhibited the proliferation of carcinoma cell lines in vitro and prolonged the life of albino mice by reducing tumor cell viability and tumor size in vivo [20]. Furthermore, the methanol crude extract of TTF fruits and four of its isolated constituents had antiproliferative activity towards drug-susceptible and multidrug-resistant cancer lines, with IC_50_ values for the crude extract and the most active compound (olean-12-en-3-*β-O*-_D_-glucopyranoside) ranging from 10.27 μg/mL (in CCRF-CEM leukemia cells) and 23.61 μg/mL (against HCT116 p53^−/−^ colon adenocarcinoma cells) and from 4.76 μM (against CCRF-CEM cells) to 12.92 μM (against HepG2 hepatocarcinoma cells) [21]. The cytotoxicity of botanicals from this plant was also reported against Jurkat leukemia cells and MCF-7 breast cancer cells [22]. The hydro-ethanolic extract of TTF had lipid-lowering, anti-inflammatory, and hypoglycemic activity, justifying its use in the management of obesity and type 2 diabetes [23]. The ethanolic extract of TTF and the aqueous extract of its bark induced renal toxicity and an increase in LDL-cholesterol, as well as liver toxicity at high doses of 400 mg/kg [24]. However, very little information is available on the toxicity of the dichloromethane-methanol extract of the fruits of this plant that previously showed cytotoxic effects [21]. The current study aimed at further evaluating the antiproliferative activity of TTF on a wider panel of resistant and metastatic lines and determining the toxicological profile of the active extract.

## Methods

### Collection and identification of plant material

Following the approval of our research project by the University of Dschang (Faculty of Science), the plant fruits were afforded in the Dschang locality in March 2021 (Subdivision of Cameroon, Western Region, 5°27′N / 10°04′E). No authorization to collect the plant sample was needed. A sample of the plant consisting of leaves and fruits was then placed in the Cameroun National Herbarium (NHC) located in Yaoundé. The sample was identified and authenticated by Mr NANA Victor as *Tetrapleura tetraptera* (Schumm. & Thonn.) Taub in comparison with the specimen of the herbarium under the voucher number 19785 SRF/Cam.

### Preparation of dichloromethane methanolic crude extract of T. tetraptera fruits

The fruits of *T. tetraptera* were dried and powdered, then macerated using dichloro-methane methanol (CH_2_Cl_2_-MeOH; 1:1) for 48 h at room temperature (RT). The macerate was further filtrated with Whatman paper No. 1. The solvent was removed from the filtrate by a rotary evaporator (BÜCHI R-200) at 65 °C to afford the crude extract (TTF). To completely remove the residual solvent, TTF was further dried in an oven at 40 °C for 6–8 h and stored in a refrigerator at 4 °C for further uses.

### Phytochemicals and chemicals

**Fig. 1:**
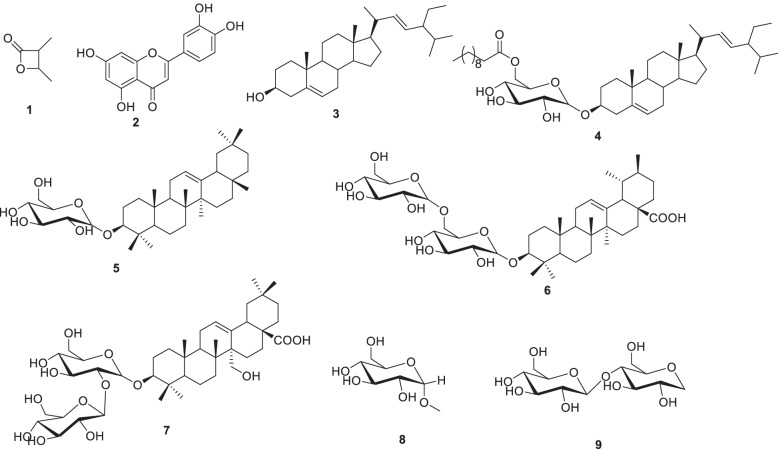
Chemical structures of compounds 1 to 9 isolated from TTF extract. 1: (3*R*, 4S)-3,4-dimethyloxetan-2-one; 2: luteolin, 3: stigmasterol; 4; 3-*O*-[6’-*O*-undecanoyl-*β*-_D_-glucopyranosyl]stigmasterol; 5: oleanan-12-en-3-*β*-*O*-_D_-glucopyranoside; 6: 3-*O*-*β*-_D_-glucopyranosyl-(1 → 6)-*β*-_D_-glucopyranosylurs-12-en-28-oic acid; 7: 3-*O*-*β*-_D_-glucopyranosyl-(1 → 3)-*β*-_D_-glucopyranosyl-27-hydroxyolean-12-ene-28-oic acid; 8: methyl-*O*-*β*-_D_-glucopyranoside; 9: *β*-_D_-fructofuranosyl-(2 → 1)-*β*-_D_-glucopyranoside

The experimented phytochemicals were: (3*R*, 4S)-3,4-dimethyloxetan-2-one (1), luteolin (2), stigmasterol (3), 3-*O*-[6’-*O*-undecanoyl-*β*-_D_-glucopyranosyl] stigmasterol (4), oleanan-12-en-3-*β*-*O*-_D_-glucopyranoside (5),3-*O*-*β*-_D_-glucopyranosyl-(1 → 6)-*β*-_D_ glucopyranosyl-12-en-28-oic acid (6), 3-*O*-*β*-_D_-glucopyranosyl-(1 → 3)-*β*-_D_-glucopyranosyl-27-hydroxyolean-12-ene-28-oic acid (7), methyl-*O*-*β*-_D_-glucopyranoside (8) and *β*-_D_-fructofuranosyl-(2 → 1)-*β*-_D_-glucopyranoside (9) (Fig. 1). Their isolation and identification from the dichloromethane-methanol (1:1) extract of TTF are given within the extra Supplementary file. Doxorubicin (purity: 98.0%) purchased from Sigma-Aldrich (Munich, Germany) was obtained from the Johannes Gutenberg University Medical Center (Mainz, Germany) [21].

### Investigated cell lines and their origin

The nine (9) malignant cell lines used in this study are originated from human and animal. Human melanoma cell lines enclosed MaMel-80a, SKMel-28 and A2058 were used as human melanoma highly lymphoid tissue metastatic cell line [25]; Mel-2a and SKMel-505 human melanoma cell line derived from the metastatic site; MV3 human melanoma cells from lung metastases in nude mice [26]; the animal originated cell line enclosed CC531 rat tumor colorectal cell line [27]; B16-F1 and B16-F10 a murine melanoma cell lines from a C57BL/6 J mouse [28–30]. SkMel-28, MaMel-80a, Mel-2A, and MV3 cell lines were provided by Prof. David Schrama (Department of Dermatology, Julius-Maximilians University, Würzburg, Germany); B16–F10 and B16–F1 murine melanoma cell lines were provided by Prof. Ugur Sahin (TRON-Translational Oncology at the University Medical Center of Johannes Gutenberg University GmbH, Mainz, Germany); CC531 rat colon adenocarcinoma cell line was purchased from CLS Cell Lines Service GmbH (Eppelheim, Germany), A2058 and SK-Mel505 human melanoma cell lines provided by Dr. Wynand P. Roos (Institute of Toxicology, Medical University Center, Mainz, Germany).

### Cytotoxicity testing of the botanical, phytochemicals, and doxorubicin by resazurin reduction assay (RRA)

To assess the actions of TTF extract, phytochemicals (1–9), and doxorubicin on cellular multiplication, the well-described RRA was applied as said earlier [31, 32] at experimental conditions like those formerly published [33–36]. Briefly, 100 μL of culture medium at a density of 1 × 10^4^ cells per well in a 96- well plate was exposed to 100 μL at concentrations starting from 0.32 – 40 μg/mL and 0.78 – 100 μM for the botanical (TTF) and phytochemicals respectively. Cells were incubated for 72 h in humidified 5% CO_2_ atmosphere at 37 °C, and the fluorescence was measured with Infinite M2000 ProTM plate reader (Tecan, Crailsheim, Germany) at 544 nm as excitation wavelength and 590 nm as emission wavelength. The sample’s concentrations needed to inhibit 50% of the cell proliferation portrayed their IC_50_ values and were deduced from a calibration curve by linear regression using Microsoft Excel 2007 [32]. The cytotoxicity results of the crude extract and the products were classified according to the scale defined by Kuete and Efferth [2], which states that for an edible plant the extract has Significant or strong cytotoxicity if IC_50_ < 50 μg/mL; moderate cytotoxicity: 50 μg/mL < IC_50_ < 200 μg/mL; low cytotoxicity: 200 μg/mL < IC_50_ < 1000 μg/mL; no cytotoxicity: IC_50_ > 1000 μg/mL and Significant or strong cytotoxicity: IC_50_ < 4 μg/mL (or IC_50_ < 10 μM); moderate cytotoxicity: 4 μg/mL < IC_50_ < 20 μg/mL (or 10 μM < IC_50_ < 50 μM); low cytotoxicity: 20 μg/mL < IC_50_ < 100 μg/mL (or 50 μM < IC_50_ < 250 μM); no cytotoxicity: IC_50_ > 100 μg/mL (or IC_50_ > 250 μM) for the chemicals.

### Experimental animals

For the toxicological study of the crude extract of TTF, young healthy males, and female *albino Wistar* rats, nulliparous and non-pregnant, aged between 1.5–2 months and weighing between 120 and 140 g, respectively, were used. The animals were obtained from the Animal House of the Department of Biochemistry, University of Dschang. They were maintained in an animal room at a temperature of 20 ± 2 °C under a standard animal room condition of 12 h light/dark cycle and fed with a standard food [37] and received drinking water ad libitum. The animals were individualized one week before the start of the experiment. The experimental protocols used for the present work were designed in concordance with the internationally accepted standard ethical guidelines for laboratory animal use and care as described in the guidelines of the European Union Institutional Ethics Committee on Animal Care (Council EEC 86/609/EEC of the 24^th^ November 1986) and were approved by the Local Ethical Committee of the Faculty of Science (University of Dschang – Cameroon). All sections of this report comply with ARRIVE Guidelines for Reporting Animal Research [38].

### Acute oral toxicity study

Acute toxicity assessment of the dichloromethane methanol crude extract of TTF was carried out according to the experimental protocol proposed by OECD Guideline 425 [39]. The crude extract was dissolved in 5% DMSO, and the volume was then adjusted to the recommended dose with distilled water. We performed the test at a single dose of 5000 mg/kg BW. We had two experimental groups of albinos female *Wistar* rats, a test group (*n* = 5) and a control group (*n* = 5). After one week of acclimatization, the animals were given a single dose of 5000 mg/kg BW of the crude extract of *T. tetraptera*. Afterward, administration of the extract by gavage, the behavior of the test animals was carefully observed for a period of 4 h to detect any signs of toxicity. After 48 h observation, the LD_50_ was determined, and the animals were then left for observation for an additional period of 14 days before being sacrificed for macroscopic observation of various organs.

### Study of sub-chronic oral toxicity

The sub-chronic toxicity was carried out on a total of 32 albino rats belonging to both genders, males, and females. The animals were divided into four groups of eight animals each, *i.e.,* four male and four female rats. The crude extract was dissolved in 5% dimethylsulfoxide (DMSO), and the volume was then adjusted with distilled water as stated above. The control group received the vehicle consisting of a 5% DMSO solution, while the other three groups constituted the test groups and respectively received a repeated administration by gavage of 250, 500, and 1000 mg/kg BW of the dichloromethane methanol crude extract of TTF for a period of 28 days. The animals were weighed every other day and the water was renewed. The food intake was carried out daily by taking the difference in mass between the starting amount of food and the amount weighed at the end of the day. The animals were carefully observed every day for any changes in clinical signs or even death. According to OECD protocol guideline 407, attention was focused on tremors, convulsions, salivation, diarrhoea, lethargy, sleep, and coma. On the last day of gavage, the rats were fasted for a period of 12 h before being sacrificed by intraperitoneal injection of ketamine. The blood was then collected by cardiac puncture and introduced into two different types of tubes, namely dry tubes for obtaining serum for the determination of biochemical parameters, and ethylenediaminetetraacetic acid (EDTA) tubes for the determination of haematological parameters. Organs such as heart, liver, kidney, and spleen were collected to determine their relative weights. Those heavily involved in xenobiotic metabolism and elimination such as the liver and kidney were used for histological sections [39].

### Hematological parameters

Blood samples collected by cardiac puncture and stored in EDTA tubes were used to perform hematological parameters by a blood count using an impedance hematology machine (QBC Auto-read Plus, United Kingdom). The parameters analyzed included red blood cells (RBC), red blood cell distribution width (CV), red blood cell distribution width (SD), hemoglobin (Hb), mean corpuscular volume (MCV); mean corpuscular hemoglobin (MCH), mean corpuscular hemoglobin concentration (MCHC), leukocytes, neutrophils, eosinophils, basophils, lymphocytes, and monocytes count, platelets (PLT), hematocrit (HCT).

### Biochemical parameters

Blood samples were collected into dried tubes and allowed to stand for 45 min at room temperature before being centrifuged at 3400 rpm for 10 min. The serum obtained was introduced into Eppendorf tubes and stored at − 25 °C and then used to evaluate the following parameters: creatinine, alanine aminotransferase (ALAT), aspartate aminotransferase (ASAT), alkaline phosphatase (ALP), total protein, total cholesterol (TC), high-density lipoprotein (HDL), low-density lipoprotein (LDL), and triglyceride (TG). These parameters were assessed using standard analytical kits (Spinreact, Spain).

### Histological analysis

For histological analysis, the liver and kidney from the sacrificed animals were first rinsed in a saline solution (0.9% NaCl) before being introduced into 3.7% formaldehyde for conservation. These tissues were subsequently dehydrated in increasing concentrations of alcohol (70%, 90%, and 100%), inserted into paraffin, and cut into sections of 4–5 μm. these paraffin Sects. (5 μm thick) were stained with hematoxylin–eosin prior to microscopic examination [40].

### Statistical analysis

Statistical analysis was performed using SPSS version 20.0 for Windows. The results were expressed as mean value ± standard deviation (SD.) and the comparisons were performed by the analysis of variance using the analysis of variance (ANOVA) test. Differences between averages of control and drug-treated groups were separated using the Waller-Duncan test. A probability value of less than 0.05 was fixed as the statistical significance criterion [41].

## Results

### Cytotoxicity

In this study where we evaluated the cytotoxicity of crude extract and phytochemicals 1–9, and doxorubicin using RRA towards 9 carcinoma cancer cell lines; it was found that, the crude TTF extract, phytochemical 5, and doxorubicin had good cytotoxic effects against the nine (9) cancer cell lines tested. The recorded IC_50_ values varied from 18.32 μM (against B16-F1 murine melanoma cells) to 36.18 μM (against SKMel-505 BRAF wildtype melanoma cells) for TTF, from 10.02 μM (towards MaMel-80a BRAF-V600E homozygous mutant melanoma cells) to 31.73 μM (against SKMel-28 BRAF-V600E homozygous mutant melanoma cells) for compound 5, and from 0.22 μM (against B16-F1 cells) to 9.39 μM (against SKMel-505 cells) for doxorubicin. Eight out of 9 cell lines tested were sensitive to compound 2 with IC_50_ values ranging from 32.20 μM (towards B16-F1 cells) to 102 μM (against SKMel-505 cells). In the sensitive cancer cell lines, the IC_50_ values ranged from 28.67 μM (against MaMel-80a melanoma cells) to 109.24 μM (towards Mel-2a melanoma cells) for compound 6; and from 29.08 μM (against MaMel-80a melanoma cells) to 79.42 μM (against A2058 melanoma cells) for compound 7. It was noticed that compounds 1, 3, 4, 8, and 9 were not active above 150 μM. The recorded IC_50_ values are summarized in Table 1.

**Table 1:** Cytotoxicity of extracts, compounds and doxorubicin towards human melanoma and other animal cancer cell lines as determined by RRA

Features and cell lines		IC_50_ values (μM)					
		**TTF**	**2**	**5**	**6**	**7**	**Doxorubicin**
BRAF-V600E homozygous mutant melanoma	MaMel-80a	30.18 ± 2.89	44.28 ± 3.29	**10.02 ± 0.21**	28.67 ± 3.12	29.08 ± 1.48	**8.66 ± 0.56**
	SKMel-28	28.23 ± 1.72	67.28 ± 5.12	31.73 ± 2.20	33.95 ± 2.53	33.11 ± 2.19	**2.14 ± 0.12**
BRAF-V600E heterozygous mutant melanoma	A2058	**19.48 ± 0.82**	78.76 ± 5.65	22.77 ± 1.72	> 150	79.42 ± 4.41	**0.29 ± 0.04**
	Mel-2a	**18.96 ± 0.69**	102.56 ± 8.94	25.45 ± 3.01	109.24 ± 8.44	> 150	**6.63 ± 0.41**
BRAF wildtype melanoma	MV3	28.72 ± 3.11	78.33 ± 6.29	23.52 ± 1.27	> 150	> 150	**7.09 ± 0.59**
	SKMel-505	36.18 ± 2.62	> 150	22.18 ± 2.01	88.74 ± 6.42	> 150	**9.39 ± 1.01**
Rat colon adenocarcinoma	CC531	**18.95 ± 1.27**	54.39 ± 3.63	**10.21 ± 0.14**	66.38 ± 4.77	54.11 ± 3.10	**0.44 ± 0.23**
Murine melanoma	B16-F1	**18.32 ± 0.96**	32.20 ± 2.17	24.08 ± 0.99	44.90 ± 5.03	69.04 ± 4.95	**0.22 ± 0.01**
	B16-F10	**20.04 ± 1.45**	41.85 ± 3.69	24.64 ± 2.56	50.12 ± 3.76	61.45 ± 5.21	**0.24 ± 0.03**

TTF: dichloromethane-methanol extract of TTF; **1:** (3*R*, 4*S*)-3,4-dimethyloxetan-2-one; **2:** luteolin; **3:** stigmasterol; **4:** 3-*O*-[6’-*O*-stearoyl-*β*-_D_-glucopyranosyl]stigmasterol; **5:** olean-12-en-3-*β*-*O*-_D_-glucopyranoside; **6:** 3-*O*-*β*-_D_-glucopyranosyl-(1 → 6)-*β*-_D-_glucopyranosylurs-12-en-28-oic acid; **7:** 3-*O*-*β*-_D_-glucopyranosyl-(1 → 3)-*β*-_D_-glucopyranosyl-27-hydroxyolean-12-en-28-oic acid; **8:** methyl-*O*-*β*-_D_-glucopyranoside; **9:** *β*-_D_-fructofuranosyl-(1 → 4)-*β*-_D_-glucopyranoside. Compounds **1**, **3**, **4**, **8** and **9** were not active, with no observable IC_50_ at up to 150 μM. In bold: significant cytotoxic effect (Boik, 2001; Brahemi et al., 2010; Kuete and Efferth, 2015); Boik, 2001; Brahemi et al., 2010)

### Acute toxicity of TTF

A single oral dose administration of 5000 mg/kg BW TTF in female rats did not cause any deaths 48 h after the administration of TTF and even during 14 days of observation. No signs of toxicity in general appearance (reduction of locomotion, stool appearance, drowsiness, salivation, reaction to noise) were observed in animals receiving this dose after 14 days of observation. Based on the OECD principle, the LD_50_ of the dichloromethane methanol extract of TTF was estimated to be higher than 5000 mg/kg.

### Sub-chronic toxicity

#### Effect of TTF on food consumption and body weight

**Fig. 2:**
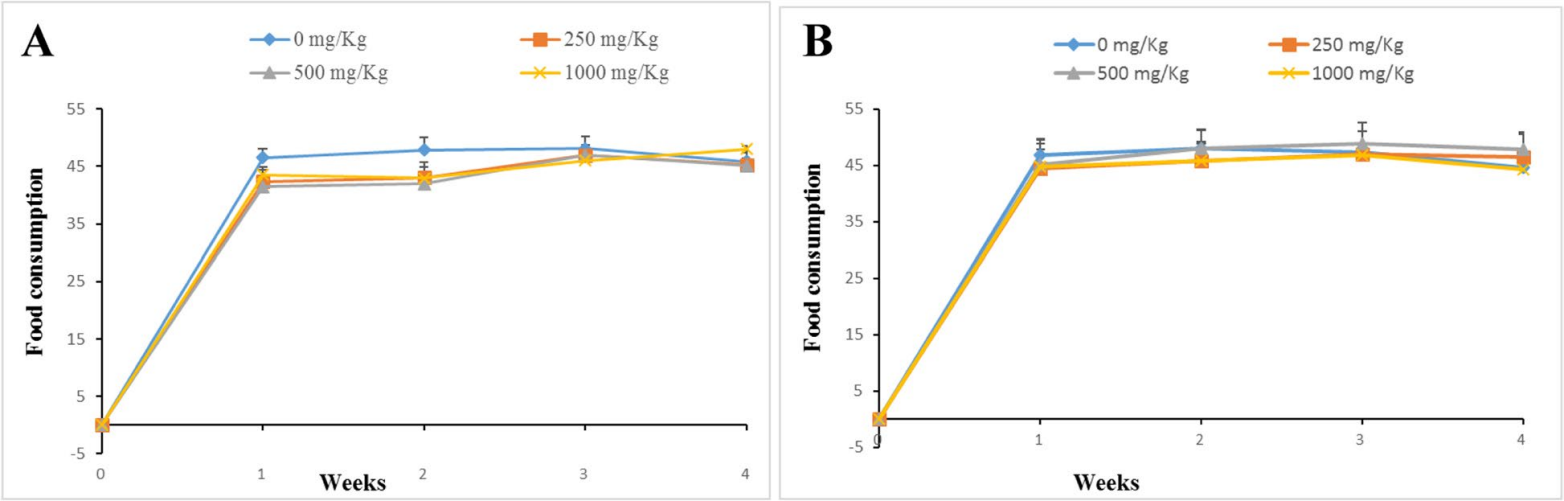
Effect of daily oral administration of TTF on food intake in female (**A**) and male (**B**) rats. Animals received TTF (250, 500, and 1000 mg/kg, BW) and vehicle (5% DMSO solution). All data are presented as mean ± SD for *n* = 4 per group

**Fig. 3:**
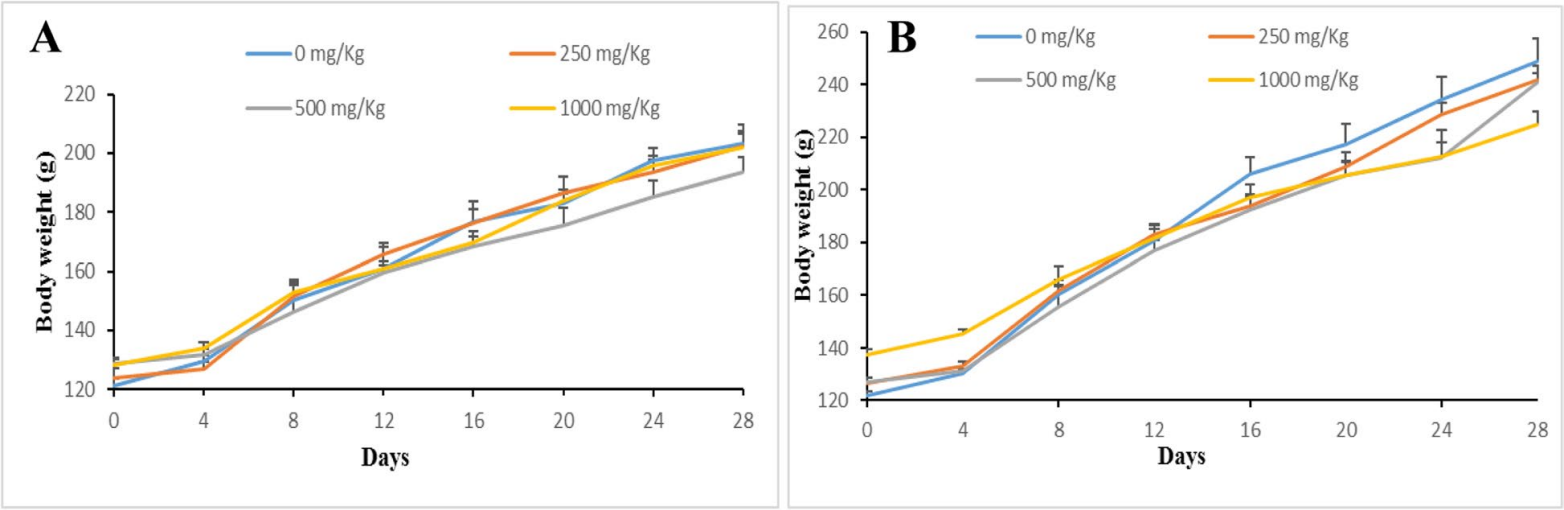
Effect of daily oral administration of TTF on body weight changes in female (**A**) and male (**B**) rats. Animals received TTF (250, 500, and 1000 mg/kg, BW) and vehicle (5% DMSO solution). All data are presented as mean ± SD for *n* = 4 per group

Neither death nor toxicity signs were observed on animals following 28 days of treatment with various doses of TTF (250, 500, 1000 mg/kg BW). Figures 2 and 3 summarise the data on the food intake and body weight evolution respectively. No significant differences (*p* ≥ 0.05) in food consumption were noted in treated and untreated males, while a significant decrease (*p* < 0.05) in food consumption was observed in treated females until the third week when no significant differences were observed. There was no weight loss in treated male and female animals and the control in this study. However, there was a small gain in body weight in animals treated at 500 and 1000 mg/kg in males and 500 mg/kg in females compared to the control.

#### Effect of TTF on relative organ weight

Table 2 summarises the relative organ weight of the liver, kidney, lung, heart, and spleen for male and female rats. No significant differences (*p* ≥ 0.05) in the relative liver, kidney, lung, and heart organ weights were observed in females for all dose groups tested compared to the control group, while a significant decrease in relative spleen weight was observed for all dose levels administered compared to the control group. A significant decrease (*p* < 0.05) in relative liver and spleen organ weights was noted at all doses in male rats compared to the control group. However, there was no significant change in the relative weights of the kidney, lung, and heart organs in male rats.

**Table 2:** Effect of different doses of *T. tetrapleura* extract on the relative weight of organs

Doses (mg/Kg)	**Female**				**Male**			
	**0**	**250**	**500**	**1000**	**0**	**250**	**500**	**1000**
Liver (g)	3.12 ± 0.09 ^ab^	3.21 ± **0.**07 ^b^	3.10 ± 0.03 ^ab^	3.09 ± 0.05 ^a^	3.39 ± 0.13^b^	3.08 ± 0.02 ^a^	3.10 ± 0.08 ^a^	3.06 ± 0.07 ^a^
Kidneys (g)	0.65 ± 0.02 ^a^	0.69 ± 0.02 ^a^	0.67 ± 0.04 ^a^	0.64 ± 0.03 ^a^	0.65 ± 0.03 ^a^	0.66 ± 0.02 ^a^	0.64 ± 0.03 ^a^	0.64 ± 0.03 ^a^
Lungs (g)	0.58 ± 0.03 ^ab^	0.62 ± 0.02 ^b^	0.6 5 ± 0.07 ^ab^	0.56 ± 0.04 ^a^	0.59 ± 0.03 ^a^	0.58 ± 0.05 ^a^	0.61 ± 0.02 ^a^	0.65 ± 0.03 ^a^
Heart (g)	0.30 ± 0.01 ^a^	0.32 ± 0.02 ^a^	0.31 ± 0.02 ^a^	0.30 ± 0.02 ^a^	0.32 ± 0.02 ^a^	0.30 ± 0.02 ^a^	0.30 ± 0.01 ^a^	0.31 ± 0.02 ^a^
Spleen (g)	0.27 ± 0.02 ^b^	0.25 ± 0.02^b^	0.22 ± 0.01 ^a^	0.21 ± 0.02 ^a^	0.30 ± 0.06^b^	0.24 ± 0.01 ^b^	0.23 ± 0.02 ^ab^	0.21 ± 0.01 ^a^

Data are expressed as mean ± SD, *n* = 4. Values in the test groups carrying the same letter as the control group in the same gender and in the same row are not significantly different according to Waller Duncan’s Multiple Comparison Test (*p* < 0.05)

#### Effect of TTF on hematological parameters

The results of the haematological parameters (Table 3) revealed an increase (*p* < 0.05) in white blood cell count at 1000 mg/kg BW. In addition, there was a significant decrease (*p* < 0.05) in the level of HGB, and HCT in female rats receiving the 1000 mg/kg dose compared to the control group. We also observed a significant decrease (*p* < 0.05) in PLT and PCT levels for all the different doses tested compared to the control group. In male rats, a significant decrease (*p* < 0.05) was observed at 1000 mg/kg for HCT and PCT levels, and at all doses tested for PLT levels. There was also a significant increase in GR and a non-significant increase in WBC compared to control.

**Table 3:** Hematological parameters in male and female rats after 28 days of administration

Sexe	**Female**				**Male**			
Dose (mg/kg)	**0**	**250**	**500**	**1000**	**0**	**250**	**500**	**1000**
WBC (10^3^/μL)	4.60 ± 0.36^ab^	4.87 ± 0.12^a^	5.30 ± 0.61^bc^	5.80 ± 0.35^c^	5.47 ± 0.31^a^	5.67 ± 0.78^a^	6.10 ± 0.66^a^	6.00 ± 0.60^a^
LY (%)	65.87 ± 1.76^ab^	64.03 ± 1.80^a^	65.50 ± 3.11^ab^	69.30 ± 1.91^b^	65.80 ± 4.39^a^	62.93 ± 2.69 ^a^	62.80 ± 5.30 ^a^	62.37 ± 3.32 ^a^
MO (%)	6.12 ± 0.55^a^	5.13 ± 0.61^a^	5 ± 0.89^a^	5.83 ± 0.70^a^	4.37 ± 0.81^ab^	5.70 ± 0.96^b^	6.35 ± 1.2^b^	3.73 ± 0.95a
GR (%)	27.80 ± 1.77^ab^	30.83 ± 1.65^b^	30.50 ± 2.69^b^	25.97 ± 0.76^a^	26.80 ± 0.30^a^	30.97 ± 1.72^b^	32.60 ± 2.78^b^	32.20 ± 1.51^b^
RBC (10^6^/μL)	8.56 ± 0.48^a^	8.66 ± 0.46^a^	8.69 ± 0.67^a^	8.25 ± 0.21^a^	9.12 ± 0.48^a^	8.70 ± 0.31^a^	8.50 ± 0.18^a^	8.94 ± 0.45^a^
HGB (g/dL)	18.30 ± 0.66^b^	18.27 ± 0.76^b^	17.70 ± 0.72^b^	16.20 ± 0.46^a^	17.53 ± 0.12^a^	17.5 ± 0.93^a^	16.50 ± 0.26^a^	17.33 ± 0.90^a^
HCT (%)	54.43 ± 1.66^b^	54.13 ± 1.56^b^	51.97 ± 2.22^ab^	47.77 ± 2.92^a^	54.57 ± 2.94^b^	51.03 ± 1.83^ab^	52.30 ± 2.46^ab^	49.47 ± 2.02^a^
MCV (fL)	60.10 ± 0.78^b^	60.43 ± 1.19^b^	59.67 ± 1.07^ab^	57.90 ± 0.79^a^	60.50 ± 1.15 ^a^	59.10 ± 1.20^a^	59.53 ± 0.65 ^a^	60.17 ± 0.80 ^a^
MCH (pg)	19.87 ± 0.31^a^	20.30 ± 0.72^a^	19.90 ± 0.61^a^	19.83 ± 0.35^a^	18.70 ± 0.52 ^a^	19.53 ± 0.75^a^	19.20 ± 0.53^a^	19.92 ± 0.78^a^
MCHC (g/dL)	33.63 ± 1.14^a^	33.10 ± 0.50^a^	34.43 ± 0.85^a^	33.53 ± 0.45^a^	31.63 ± 1.12 ^a^	32.53 ± 1.07 ^a^	31.90 ± 0.70 ^a^	33.70 ± 1.00 ^a^
RDWCV (%)	15.87 ± 0.21^a^	16.97 ± 1.53^a^	16.60 ± 0.61^a^	16.83 ± 0.93^a^	16.47 ± 0.32^a^	16.53 ± 0.15^a^	15.90 ± 0.56^a^	17.23 ± 1.50^a^
RDWSCD (fL)	36.53 ± 1.64^a^	41.3 ± 2.18^b^	38.43 ± 0.55^a^	41.17 ± 1.50^b^	40.10 ± 0.36 ^a^	39.50 ± 1.37 ^a^	37.87 ± 1.76 ^a^	40.73 ± 4.62 ^a^
PLT (10^3^/μL)	888.67 ± 21.46^b^	730.33 ± 32.15^a^	717.33 ± 25.74^a^	727.33 ± 44.77^a^	666.33 ± 29.16^b^	609 ± 41.8^ab^	597.67 ± 24.38^a^	569.67 ± 19.14^a^
PCT (%)	0.61 ± 0.02^b^	0.46 ± 0.06^a^	0.49 ± 0.02^a^	0.50 ± 0.03^a^	0.49 ± 0.03^b^	0.43 ± 0.05^ab^	0.46 ± 0.02^b^	0.37 ± 0.02a
PDW (fL)	17.57 ± 0.23^a^	17.33 ± 0.55^a^	17.00 ± 0.53^a^	17.10 ± 0.75^a^	17.03 ± 0.47^ab^	18 ± 0.50^b^	16.73 ± 0.29^a^	17.63 ± 0.29^b^
MPV (fL)	6.77 ± 0.12^a^	7.02 ± 0.27^a^	6.83 ± 0.12^a^	6.83 ± 0.12^a^	6.90 ± 00 ^a^	6.97 ± 0.31 ^a^	6.60 ± 0.17 ^a^	6.90 ± 0.20 ^a^

Data are expressed as mean ± SD, *n* = 4. Values in the test groups carrying the same letter as the control group in the same-sex and in the same row are not significantly different according to Waller Duncan’s Multiple Comparison Test (*p* < 0.05). *WBCs* White blood cells, *RBCs* Red blood cells, *HCT* Hematocrit, *PLT* Platelets, *HGB* Haemoglobin, *MCH* Mean corpuscular haemoglobin, *MCHC* Mean corpuscular haemoglobin concentration, *MCV* Mean corpuscular volume, *Gran* Granulocytes, *Lym* Lymphocytes, *MO* Monocytes, *RDWCV* Red blood cells distribution width CV, *RDWSD* Red blood cells distribution width SD, *PCT* Plateletcrit, *MPV* Mean platelet volume, *PDW* Platelet distribution width

#### Effect of TTF on urinary biochemical parameters

The results of the urinary and serum biochemical parameters of rats treated with methanol extract of TTF are presented in Table 4. The extract did not affect urinary protein levels in either sex. There was a significant decrease (*p* < 0.05) in urinary creatinine from the 500 mg/kg dose onwards in female rats whereas no significant difference was observed in males compared to the control group. In both sexes, there was a significant increase (*p* < 0.05) in urine urea at 1000 mg/kg in females and 500 mg/kg in males compared to the control groups.

**Table 4:** Urinary biochemical parameters in males and females after 28 days of administration

Sex Doses (mg/Kg)	**Female**				**Male**			
	**0**	**250**	**500**	**1000**	**0**	**250**	**500**	**1000**
Creatinine (mg/dL)	2.85 ± 0.13^b^	2.62 ± 0.16^b^	2.04 ± 0.06^a^	2.04 ± 0.06^a^	4.11 ± 0.04^b^	3.86 ± 0.05^a^	3.68 ± 0.04^a^	3.49 ± 0.06^a^
Urea (mg/dL)	107.23 ± 4.26^a^	104.07 ± 3.66^a^	108.43 ± 2.25^a^	130.12 ± 2.51^b^	106.93 ± 2.67^a^	113.41 ± 4.03^a^	131.93 ± 4.69^b^	133.73 ± 1.77^b^
Total Protein (g/dL)	0.79 ± 0.05^a^	0.79 ± 0.05^a^	0.85 ± 0.05^a^	0.83 ± 0.06^a^	0.82 ± 0.03^a^	0.86 ± 0.06^a^	0.85 ± 0.05^a^	0.80 ± 0.06^a^

Data are expressed as mean ± SD, *n* = 4. Values in the test groups carrying the same letter as the control group in the same gender and in the same row are not significantly different according to Waller Duncan’s Multiple Comparison Test (*p* < 0.05)

#### Effect of TTF on serum biochemical parameters

Table 5 shows the results of serum biochemical parameters of the animals in the control groups and those that received different TTF doses (250, 500, and 1000 mg/kg BW). In both male and female rats, after prolonged administration, a significant decrease (*p* < 0.05) in the values of transaminase activity (ALAT and ASAT), alkaline phosphatase, and total protein concentration were observed for all doses administered in comparison with the control group. In addition, a significant increase (*p* < 0.05) in urea levels was observed in male rats at all doses tested but was only observed in female rats at doses above 1000 mg/kg. A significant decrease in serum creatinine was observed in male rats at all doses tested compared to the control group (*p* < 0.05).

**Table 5:** Serum biochemical parameters in males and females after 28 days of administration

Doses (mg/Kg)	**Female**				**Male**			
	**0**	**250**	**500**	**1000**	**0**	**250**	**500**	**1000**
Creatinine (mg/dL)	0.78 ± 0.02^a^	0.78 ± 0.05^a^	0.85 ± 0.05^a^	0.84 ± 0.03^a^	1.44 ± 0.03^c^	1.33 ± 0.02^b^	1.27 ± 0.01^a^	1.28 ± 0.02^a^
Urea (mg/dL)	71.23 ± 3.35^a^	71.23 ± 1.58^a^	73.80 ± 7.27^a^	87.35 ± 2.56^b^	62.35 ± 0.78^a^	70.48 ± 0.98^b^	72.29 ± 2.25^bc^	74.25 ± 1.86^c^
ASAT (U/L)	107.18 ± 2.31^b^	106.96 ± 1.49^b^	94.62 ± 2.57^a^	97.56 ± 1.82^a^	105.43 ± 2.31^c^	84.43 ± 3.23^b^	73.71 ± 2.61^a^	73.06 ± 1.12^a^
ALAT (U/L)	61.19 ± 1.82^b^	57.31 ± 1.13^a^	57.75 ± 1.43^a^	57.09 ± 1.31^a^	65.19 ± 2.08^c^	57.53 ± 1.66^b^	53.81 ± 2.53^a^	54.03 ± 1.49^a^
PAL (U/L)	363.43 ± 3.12^d^	308.26 ± 1.49^c^	261.29 ± 3.12^b^	200.18 ± 1.75^a^	420.43 ± 2.35^c^	376.66 ± 2.35^b^	373.46 ± 3.12^b^	352.94 ± 2.35^a^
Total Protein (g/dL)	8.70 ± 0.29^b^	7.66 ± 0.22^a^	7.37 ± 0.58^a^	7.02 ± 0.42^a^	10.31 ± 0.07^b^	9.13 ± 0.85^a^	8.38 ± 0.70^a^	7.92 ± 0.59^a^

Data are expressed as mean ± SD, *n* = 4. Values in the test groups carrying the same letter as the control group in the same gender and in the same row are not significantly different according to Waller Duncan’s Multiple Comparison Test (*p* < 0.05). *ASAT* Aspartate amino transferase, *ALAT* Alanine amino transferase, *PAL* Phosphatase alkaline

#### Effect of TTF on lipid profile of male and female rats after 28 days of oral treatment

All the lipid profile values obtained from the serum of the control and test groups at different doses (250, 500, and 1000 mg/kg) after 28 days of oral administration are presented in Table 6. A significant decrease (*p* < 0.05) in triacylglycerol (TAG) was observed in female rats from the dose of 500 mg/kg. LDL cholesterol varied significantly, by decreasing (*p* < 0.05) at all doses tested in both males and females compared to the control group. A significant increase (*p* < 0.05) in total cholesterol and HDL cholesterol values was observed in both males and females compared to the control group for all different doses tested.

**Table 6:** Lipid profile parameters in females and males after 28 days of administration

Doses (mg/Kg)	**Female**				**Male**			
	**0**	**250**	**500**	**1000**	**0**	**250**	**500**	**1000**
TAG (mg/kg)	62.45 ± 2.25^b^	62.83 ± 1.29^b^	57.23 ± 3.09^a^	50.94 ± 3.96^a^	67.17 ± 3.08^a^	71.13 ± 3.28^a^	69.81 ± 2.35^a^	65.85 ± 3.04^a^
T-CHOL (mg/Kg)	82.60 ± 1.77 ^a^	90.54 ± 1.57 ^b^	95.96 ± 2.89^c^	103.78 ± 3.2^d^	88.78 ± 1.30^a^	93.82 ± 3.80^ab^	97.23 ± 4.59^b^	112.86 ± 2.58^c^
HDL (mg/Kg)	52.71 ± 2.12 ^a^	62.04 ± 0.71^b^	68.98 ± 3.01^c^	81.34 ± 3.17^d^	53.68 ± 1.94^a^	66.33 ± 2.78^b^	69.99 ± 4.84^b^	83.23 ± 2.97^c^
LDL (mg/Kg)	18.70 ± 1.14^c^	15.93 ± 1.30^b^	15.51 ± 1.38^b^	12.26 ± 1.03^a^	21.66 ± 0.84^b^	13.26 ± 2.27^a^	13.28 ± 1.28^a^	16.46 ± 2.78^a^

Data are expressed as mean ± SD, *n* = 4. Values in the test groups carrying the same letter as the control group in the same-sex and in the same row are not significantly different according to Waller Duncan’s Multiple Comparison Test (*p* < 0.05). *TAG* Triacylglycerol, *T-CHOL* Total cholesterol, *HDL* High density lipoprorotein, *LDL* Low density lipoprotein

#### Histological sections of the liver and kidneys

**Fig. 4:**
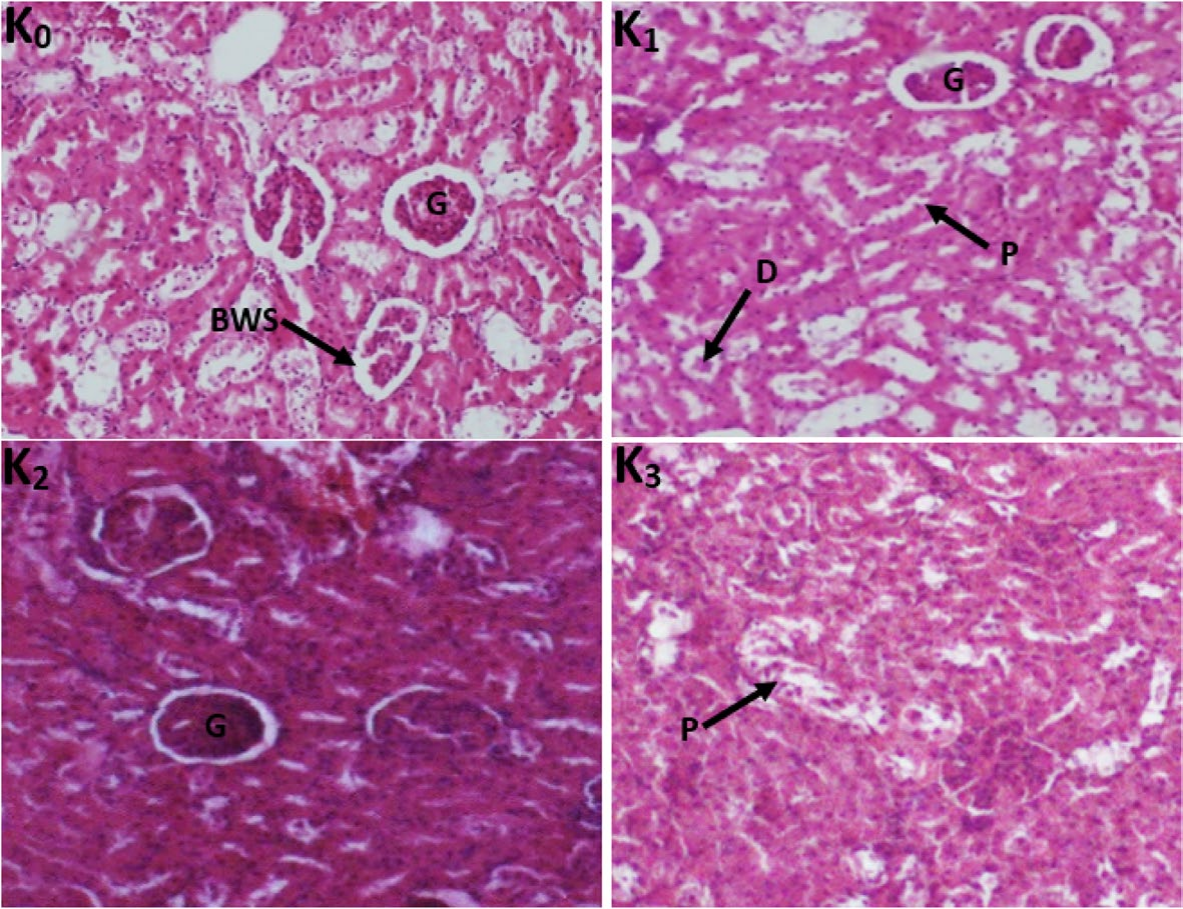
Photomicrograph of the kidney section showing the effect of TTF in 28-day subacute toxicity study in female rats (400 ×). (I_0_) control; (I_1_) Wistar strain rats treated with 250 mg/kg TTF extract; (I_2_) Wistar strain rats treated with 500 mg/kg TTF extract; (I_3_) Wistar strain rats treated with 1000 mg/kg TTF extract; G: glomerulus, BWS: Bowman space, DCT: distal convoluted tubule, PTC: proximal convoluted tubule

**Fig. 5:**
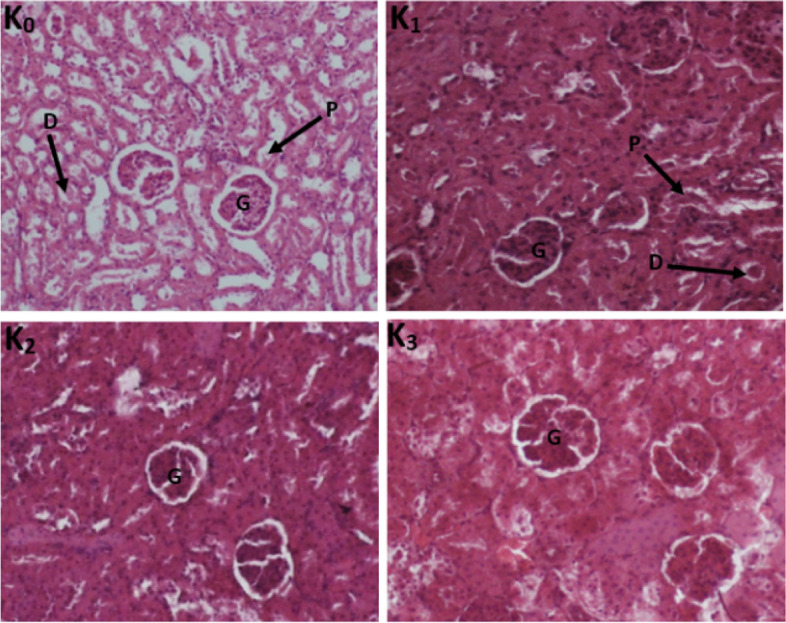
Photomicrograph of the kidney section showing the effect of TTF in 28-day subacute toxicity study in male rats (400 ×). (I_0_) control; (I_1_) Wistar strain rats treated with 250 mg/kg TTF extract; (I_2_) Wistar strain rats treated with 500 mg/kg T; (I_3_) Wistar strain rats treated with 1000 mg/kg TTF extract; G: glomerulus, BWS: Bowman space, DCT: distal convoluted tubule, PTC: proximal convoluted tubule

**Fig. 6:**
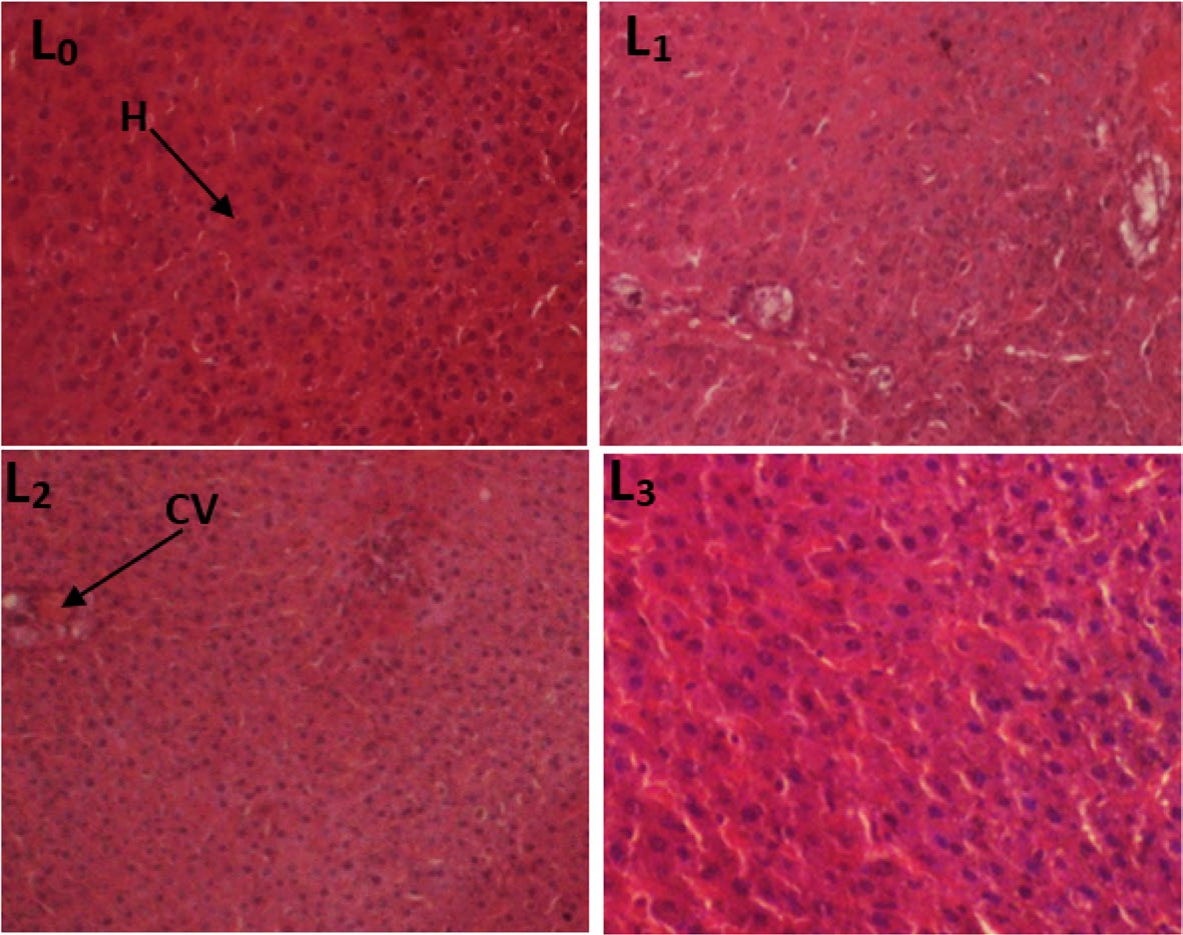
Photomicrograph of the liver section showing the effect of TTF in 28-day subacute toxicity study in female rats (400 ×). (L_0_) control; (L_1_) Wistar strain rats treated with 250 mg/kg TTF extract; (L_2_) Wistar strain rats treated with 500 mg/kg TTF extract; (L_3_) Wistar strain rats treated with 1000 mg/kg TTF extract, H: hepatocytes, C: Centrolobular vein

**Fig. 7:**
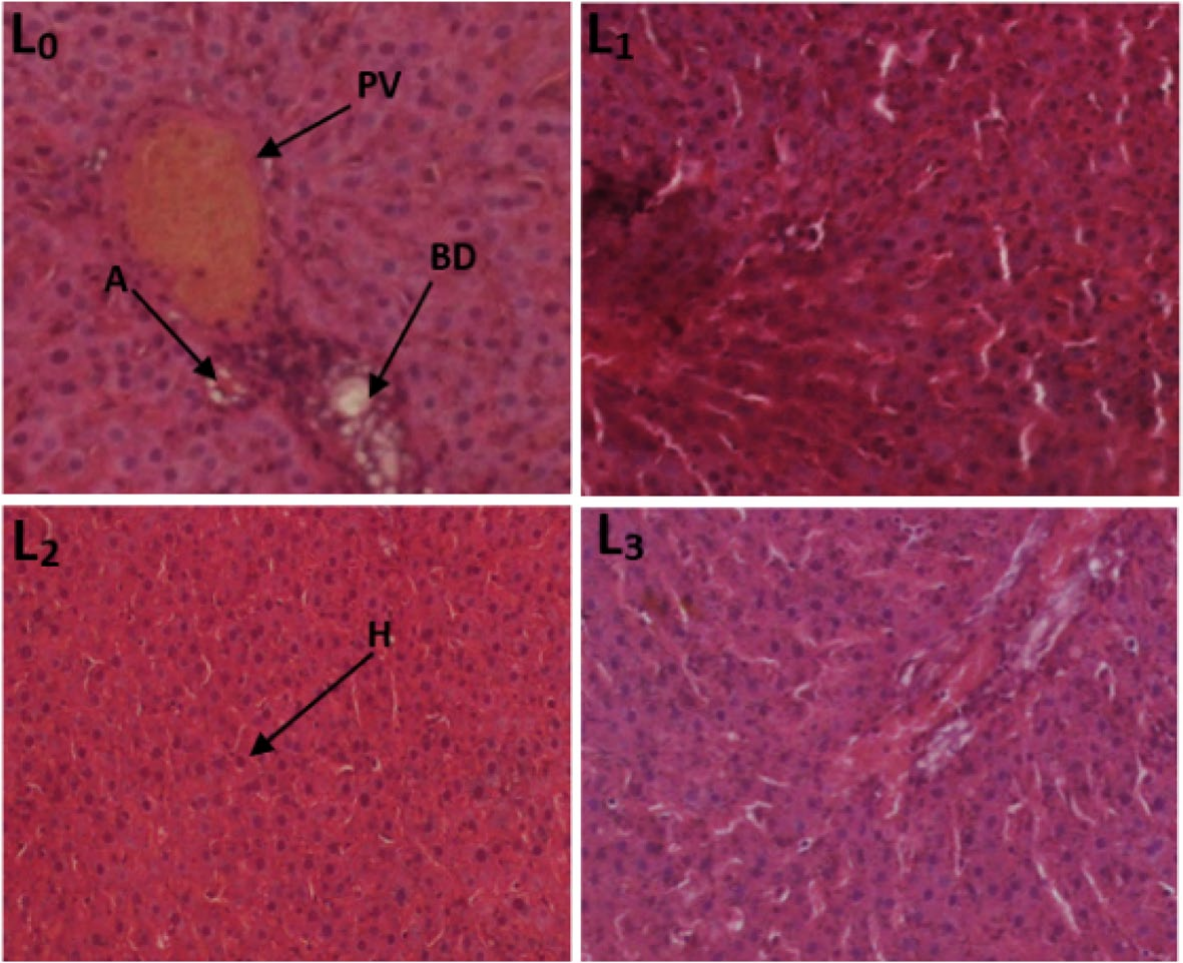
Photomicrograph of the liver section showing the effect of TTF in 28-day subacute toxicity study in male rats. (L_0_): control group; (L_1_): 250 mg/kg; (L_2_): 500 mg/kg and (L_3_): 1000 mg/kg. Indicators: PV: portal space; A: hepatic artery H: hepatocytes; (BD): bile duct

Histopathological examinations were performed on the liver and the kidney to assess organ damage. The normal rat liver has a microscopic architecture structured in hexagonal lobules and acini. The hexagonal lobules are centered on the central vein (CV) with surrounding hepatocyte cords and have a portal triad containing branches of the hepatic artery (HA), the bile duct (BD), and the portal vein (PV). The photomicrograph of the liver section of normal rats shows the glomeruli which are surrounded by proximal convoluted tubes consisting of cuboid cells with a brush border and distal convoluted tubes consisting of round cells. The kidney of treated rats showed normal glomeruli and there was no necrosis of tubular epithelium either in female (Fig. 4) or male (Fig. 5) treated rats. Adverse effects were neither found in the liver of female (Fig. 6) nor male (Fig. 7) treated rats compared to the control group.

## Discussion

According to the new Globocan estimates, the global cancer burden has increased to 19.3 million cases and 10 million cancer deaths in 2020 [1]. Cancer treatments including chemotherapy, immunotherapy, and hormone therapy have significantly improved patient survival in recent years. Although these drugs have a significant impact on reducing the mortality of cancer patients, they have numerous side effects including nephrotoxicity, hepatotoxicity, and cardiotoxicity, etc. [42, 43]. This toxicity forces a reduction of the doses leading inevitably to the emergence of multidrug resistance against conventional anticancer drugs. Therefore, the challenge in the fight against cancer is to find anticancer drugs with acceptable toxicity, and capable not only of eliminating sensitive cancers but above all of defeating those with resistant and metastatic phenotypes. It is important to underline the use of dichlomethane-methanol as an extraction solvent in the present study. Like the phytochemical study of the methanol extract, that of the *Tetrapleura tetraptera* fruits dichloromethane-methanol (1:1) extract revealed the presence of alkaloids, saponins, tannins, flavonoids, reducing sugars, glycosides, terpenoids, phenols, steroids, and anthraquinones [29, 44, 45]. However, there are several classes of isolated secondary metabolites. Thus, compounds such as N-acetylglycoside of oleanolic acid, oleanan-12-en-3-*β*-*O*-_D_-glucopyranoside, and 3-*O-β*-_D_-glucopyranosyl (1 → 6)-*β*-_D_-glucopyranosyl-12-en-28-oic acid isolated from the dichloromethane-methanol extract and absent from the methanol extract shown very good cytotoxic activities [29]. Indeed, a preliminary study on the methanol extract showed a lower cytotoxic activity, justifying that the dichloromethane-methanol mixture (1:1) would make a better extraction solvent for *Tetrapleura tetraptera* fruits in the fight against cancer [9]. It has been shown in the present study that the crude extract of TTF had a 100% spectrum of activity on human and animal metastatic colorectal and melanoma cancer cell lines with significant IC_50_ values below 20 μg/mL on more than 50% of the tested cell lines [2]. This result is justified by its composition in secondary metabolites rich in molecules with proven anticancer activity as demonstrated in a previous study [46]. Similarly, oleanolic acid *N*-acetylglycoside which is a compound previously isolated from the dichloromethane methanol extract of the fruits of this plant exhibited a 100% antiproliferative activity spectrum on a panel of 18 resistant and metastatic cancer cell lines with IC_50_ values ranging from 3.18 μM (CCRF-CEM leukemia cells) to 9.56 μM (HepG2 hepatocarcinoma cells) [46]. In an earlier reported study, TTF induced apoptosis in CCRF-CEM cells, mediated by the alteration of the mitochondrial membrane potential (MMP) and increased production of reactive oxygen species (ROS) in CCRF-CEM cells, whilst one of its constituents, olean-12-en-3-*β-O*-_D_-glucopyranoside also induced apoptosis mediated by caspases activation, MMP alteration and increased ROS production [46]. TTF was also cytotoxic towards MCF-7 cells and Jurkat cells with IC_50_ values of 380.12 μg/mL and 340.47 μg/mL, respectively [22]. These data are weaker than those obtained in the present work, probably showing that dichlomethane-methanol might be the best extractive solvent for the cytotoxic components of the plant. Herein, the recorded activity spectra were 100% for compound 5, 88.88% for compound 2, 77.77% for compound 6, and 66.66% for compound 7. These results are an indication that these phytochemicals are potential anticancer agents. Regarding the interesting antiproliferative activity of the extract and its isolates, further toxicity studies are planned in an animal model.

Although plants are traditionally used in the management of several diseases, their toxicity is not excluded. Many previous works related to the study of the toxicity of plant extracts demonstrated their aggressiveness towards many organs altering their physiological functions [47, 48]. It has also been reported that repeated and prolonged administration of methanol extract of some spices such as *Piper capense* and *Imperata cylindrica* at doses higher than 500 mg/kg BW provoked adverse effects on liver and kidney function [49, 50]. In the present study, the toxicity of dichloromethane methanol extract of TTF after its acute and sub-chronic administration was evaluated in *Wistar* strain albino rats. The results of the acute study indicated that oral administration of TTF at a single dose of 5000 mg/kg did not induce any mortality and no signs of toxicity in rats. This suggested an LD_50_ value greater than 5000 mg/kg indicating that TTF is practically non-toxic [50]. Moreover, according to the OECD (2008a), orally ingested substances with an LD_50_ value > 5000 mg/kg are considered relatively safe. This result corroborates those of many other studies which also demonstrated that the LD_50_ following a single dose of the extracts of certain spices was higher than 5000 mg/kg BW [49, 50]. The work of Dongmo et al. [24]. on the aqueous extract of the bark of *T. tetraptera* also led to a similar result. On the other hand, the study of the acute toxicity of the ethanolic extract of the fruits of this plant on a fish culture showed a mortality of 50% after 54 h of exposure to a concentration of 45 mg/L of the extract [51]. The difference observed compared to our study could be justified by the difference in the extraction solvent used and the exposure time which was relatively shorter in our study where the animals received a single dose by gavage.

Repeated administration over a period of 28 days did not cause mortality or signs of systemic toxicity in the test animals. After analysis of the results of Table 2 summarising the relative weight of the organs, no significant difference was observed in the relative organ weights of the treated animals compared to the control group. This would be justified by the absence of compounds in the dichloromethane methanol extract of TTF that cause excessive organ growth. It is important to note that some plant extracts can have hypertrophic effects on organs exposing the organism to develop cancers [52]. Daily administration for a period of 28 days of TTF showed a significant increase in white blood cells.

The hematopoietic system is known to be highly sensitive to toxic substances and is also a significant indicator of physiological and pathological conditions in humans and animals [53]. As shown in Table 3 in this study, the dichloromethane methanol extract of TTF significantly (*p* < 0.05) increased the levels of white blood cells (WBC) in females receiving the highest dose compared to the control group. This could be attributed to the boosting capacity of the immune system of the test animals by the bioactive molecules in the extract [54]. This is not in agreement with other works that have shown a decrease in the level of white blood cells following the treatment of animals with ethanolic extract of the fruits of this plant [55]. This could be explained by the difference in the extraction solvent which was dichloromethane methanol in the present work. The non-significant effect of the extract on the RBC may be an indication that the balance between the rate of production (erythropoiesis) and destruction of the blood corpuscles was not altered. Therefore, the significant decrease (*p* < 0.05) in HGB, PCT, and MCH levels in females treated with the highest dose could mean that the incorporation of hemoglobin into the red blood cells and the morphology of the red blood cells were altered [56]. The platelet counts and platelet blood volume (PCT) also decreased (*p* < 0.05) appreciably at all treated dosages of the extract in both females and males. This could be explained by the ability of the extract to prevent the development and fragmentation of megakaryocytes in the bone marrow. The liver plays a major role in the detoxification and excretion of many endogenous and exogenous compounds; any damage or impairment of its functions can have many consequences for human and animal health [57]. Liver damage is associated with cellular necrosis, increased tissue lipid peroxidation, and depletion of reduced glutathione levels. In addition, serum levels of many biochemical markers such as transaminases, alkaline phosphatase, triglycerides, and cholesterol are elevated in liver disease [57]. The evaluation of liver function in this study through serum biochemical parameters such as ALT, ASAT, PAL, and total protein summarized in Table 5 showed a significant decrease (*p* < 0.05) in all these parameters and at all doses tested. Based on these results, TTF would not affect the proper functioning of the liver because any hepatocellular injury would have resulted in a serum increase in ALT and AST levels [58]. This could be justified by a hepatoprotective effect of the extract due to its phenolic compounds which play an antioxidant role in preventing the peroxidation of membrane lipids [59, 60].

The primary function of the kidneys is to eliminate the toxic waste produced by the normal functioning of the body and transported by the blood. Indeed, many previous works strongly confirm the ability of plant extracts to act in the kidney as powerful scavengers of free radicals; preventing their toxic effects on lipid peroxidation responsible for the upward variation of biochemical parameters such as creatinine and urea via the destruction of membranes [61, 62]. Table 4 of this study on urinary parameters, jointly showed a significant decrease (*p* < 0.05) in urinary and serum creatinine in male rats at all doses, and from dose 500 mg/kg in females for serum creatinine. This could be justified by the nephroprotective effect of TTF extract through the antioxidant capacity of some of its constituents such as flavonoids and total phenols which would prevent membrane lysis [63]. On the contrary, a significant increase (*p* < 0.05) in urinary and serum urea was found in male rats from 500 mg/kg onwards and at all doses tested. This could notify a toxic effect of the extract from the dose of 500 mg/kg. This result is in contradiction with that of other works confirming the protective effect of methanol extract of TTF on kidney cells by inhibition of xanthine oxidase responsible for lipid peroxidation and membrane destabilization [61]. The lipid profile is a set of parameters among which LDL-cholesterol, total cholesterol, and triglycerides are those that are mainly involved in the risk factors of cardiovascular diseases [64]. The significant decrease (*p* < 0.05) in triacylglycerol, LDL as well as the increase in HDL and total cholesterol levels could be explained by the ability of the different components of TTF extract to improve the catabolism of cholesterol into bile acids [65]. Indeed, many previous works have demonstrated the lipid-lowering and anti-obesity potential of *T. tetraptera* fruit extracts [23, 66].

## Conclusion

This work aimed to demonstrate the antiproliferative activity towards human and animal cell lines of the dichloromethane methanol extract of TTF and its isolated compounds and to evaluate the toxicological profile of the extract on albino rats. The crude extract and the tested compounds, especially 5 showed cytotoxic activity toward human and animal carcinoma cell lines. The acute toxicity results of TTF suggested that the dichloromethane-methanol extract of TTF is almost nontoxic. Nonetheless, it should be taken with caution during a sub-chronic administration to avoid renal damage.

## Supplementary Information

**Additional file 1**.

**Additional file 2**.

## Data Availability

All data generated or analysed during this study are included in this published article and its supplementary information files.

## Abbreviations

ALAT: Alanine aminotransferase
ALP: Alkaline phosphatase
ASAT: Aspartate aminotransferase
BW: Body weight
DMSO: Dimethylsulfoxide
EDTA: Ethylenediaminetetraacetic acid
Hb: Hemoglobin
HCT: Hematocrit
HDL: High-density lipoprotein
LD_50_: 50% Lethal dose
LDL: Low-density lipoprotein
MCH: Mean corpuscular hemoglobin
MCH: Mean corpuscular hemoglobin
MCHC: Mean corpuscular hemoglobin concentration
MCHC: Mean corpuscular hemoglobin concentration
MCV: Mean corpuscular volume
MCV: Mean corpuscular volume
OECD: Organisation for Economic Cooperation, and Development
PLT: Platelets
RBC: Red blood cells
RRA: Resazurin reduction assay
TC: Total cholesterol
TG: Triglyceride
TTF: Extract of *T. tetraptera* fruits
