# Supplementary material for: Cytotoxicity, acute and sub-chronic toxicities of the fruit extract of Tetrapleura tetraptera (Schumm. & Thonn.) Taub. (Fabaceae)
Source: BMC Complement Med Ther. 2022 Jul 4;22:178. doi: 10.1186/s12906-022-03659-1 (PMC9252075; doi:10.1186/s12906-022-03659-1)
Supplement: Supplementary file 1 — Additional file 1. [file 12906_2022_3659_MOESM1_ESM.docx]

Cytotoxicity, acute and sub-chronic toxicities of botanical from the fruits *Tetrapleura tetraptera (Schumm. & Thonn.) Taub.* (Fabaceae)

Idrios N. Bonsou^1^, Armelle T. Mbaveng^1, 2*^, Gaëlle S. Nguenang^1^, Godloves F. Chi^3^ and Victor Kuete^1, 2***^, Thomas Efferth^2***^

^1^*Department of Biochemistry, Faculty of Science, University of Dschang, Dschang, Cameroon;*

*^2^Department of Pharmaceutical Biology, Institute of Pharmacy and Biochemistry, University of Mainz, Staudinger Weg 5, 55128 Mainz, Germany;*

^3^*Department of Chemistry, Faculty of Science University of Buea, Buea, Cameroon*

**Corresponding authors:**

** E-mail:* [*armbatsa@yahoo.fr*](mailto:armbatsa@yahoo.fr)*; (Prof. Dr. Armelle T. Mbaveng); ** E-mail:* [*kuetevictor@yahoo.fr*](mailto:kuetevictor@yahoo.fr)*; (Prof. Dr. Victor Kuete); ** *E-mail:* [*efferth@uni-mainz.de*](mailto:efferth@uni-mainz.de) *(Prof. Dr. Thomas Efferth)*

**Other author’s addresses**

*Idrios N. Bonsou:* [*Bonichrist89@yahoo.com*](mailto:Bonichrist89@yahoo.com%20)

*Gaëlle S. Nguenang:* [*sgaelle78@yahoo.com*](mailto:sgaelle78@yahoo.com)

*Godloves F. Chi:* [*chigodloves@yahoo.com*](mailto:chigodloves@yahoo.com)*;*

***Supplementary materials***

***S1:*** Isolation of phytochemicals from the crude extract of the fruits of *Tetrapleura tetraptera****;*** S2: Physical properties and NMR data of compounds 1 – 9***;*** Figure S1: HRESIMS of compound **1**; Figure S2: ^1^H NMR spectrum (400 MHz, MeOD) of compound **1**; Figure S3: ^13^C NMR spectrum (100 MHz, DMSO) of compound **1**; Figure S4: COSY spectrum of compound **1**; Figure S5: HSQC spectrum of compound **1**; Figure S6: HMBC spectrum of compound **1;** Figure S7: ESIMS spectrum of compound **2**; Figure S8: ^1^H NMR spectrum (600 MHz, DMSO) of compound **2**; Figure S9: ^13^C NMR spectrum (150 MHz, DMSO) of compound **2**; Figure S10: ^1^H NMR spectrum (600 MHz, CDCl_3_) of compound **3**; Figure S11: ^13^C NMR spectrum (150 MHz, CDCl_3_) of compound **3;** Figure S12: ^1^H NMR (300 MHz, CDCl_3_) spectrum of compound **4**; Figure S13: ^13^C NMR (75 MHz, CDCl_3_) spectrum of compound **4**; Figure S13: ^13^C APT NMR spectrum (75 MHz, CDCl_3_) of compound **4**; Figure S15: ^1^H NMR spectrum (400 MHz, MeOD) of compound **5**; Figure S16: ^13^C NMR spectrum (100 MHz, MeOD) of compound **5**; Figure S17: ^1^H NMR spectrum (400 MHz, MeOD) of compound **6**; Figure S18: ^13^C NMR spectrum (100 MHz, MeOD) of compound **6**; Figure S19: ^1^H NMR spectrum (400 MHz, MeOD) of compound **7**; Figure S20: ^13^C NMR spectrum (100 MHz, MeOD) of compound **7**; Figure S21: HSQC spectrum of compound **7**; Figure S22: ^1^H NMR spectrum (400 MHz, MeOD) of compound **8**; Figure S23: ^13^C NMR spectrum (100 MHz, MeOD) of compound **8**; Figure S24: ^1^H NMR spectrum (400 MHz, MeOD) of compound **9**; Figure S25: ^13^C NMR spectrum (100 MHz, MeOD) of compound **9**; Figure S26: TLC profiles for Hexane and Ethyl Acetate extracts; Figure S27: TLC profiles for Methanol extract.

***S1. Isolation of phytochemicals from the crude extract of the fruits of Tetrapleura tetraptera***

Part of TTF (179 g) was partitioned by a solid liquid process into hexane (Hex; 4.0 g), ethyl acetate (EtOAc; 18.3 g), MeOH (TTFb; 72.03 g) fractions (frs). Based on thin layer chromatography (TLC) profiles, the Hex and EtOAc fractions were pooled to give 22.3 g of a new fraction (TTFa). The TTFa was adsorbed on 30 g silica gel 60 (63-43 μm Merck, particle size between 0.043 and 0.063 mm in diameter and porosity 230-400 mesh ASTM). Dried silica gel-adsorbed TTFa was subjected to silica gel column chromatography using an increasing gradient of EtOAc in Hex. A total of 200 fractions of 100 mL each [(Hex: frs 1 - 15, Hex-EtOAc 5%: frs 16 - 43, Hex-EtOAc 10%: frs 44 - 60, Hex-EtOAc 15%: frs 72 - 121, Hex-EtOAc 20%: frs 122 - 142, Hex-EtOAc 25%: frs 143 - 155, Hex-EtOAc 30%: frs 156 - 165, Hex-EtOAc 75%: frs 166 - 187, EtOAc 100%: frs 188 - 200] were collected and combined on the basis of their TLC profiles. Compounds **3** (37 mg) and **4** (76 mg) were obtained from fractions 50-54 and 172-183, respectively, by washing with Hex-EtOAc mixtures and filtration. The MeOH fraction (TTFb; 72.63 g) dissolved in MeOH and adsorbed on 70.23 g of fine silica gel 60 was subjected to column chromatography, using 450 g silica gel 60 (63-43 μm Merck), with increasing gradient of MeOH in CHCl_3_. A total of 200 fractions of 200 mL each [(CHCl_3_: frs 1 - 8, CHCl_3_-MeOH 2.5%: frs 9 - 22, CHCl_3_-MeOH 5%: frs 23 - 40, CHCl_3_-MeOH 7.5%: frs 41 - 50, CHCl_3_-MeOH 10%: frs 51 - 100, CHCl_3_-MeOH 15%: frs 101 - 154, CHCl_3_-MeOH 20%: frs 155 - 169, CHCl_3_-MeOH 25%: frs 170 - 184, CHCl_3_-MeOH 30%: frs 185 - 200)] were collected and combined according to their TLC profiles into TTFb_1_ (frs 1-50), TTFb_2_ (frs 51-107), TTFb_3_ (frs 108-123) and TFbt_4_ (frs 124-200). TTFb1 obtained in very low amount (76 mg) was not further processed. Fraction TTFb2 (1.86 g) was chromatographed on silica gel column using CHCl_3_-MeOH (95:5), 100 sub-frs of 15 mL each were collected and pooled using TLC profiles. Compounds **1** (5.3 mg) and **5** (6.8 mg) were isolated from sub-frs 32-44 & 48-56 respectively. Fraction TTFb_3_ (2.5 g) was applied to a silica gel column and eluted with CHCl_3_-MeOH (90:10); 70 sub-fractions of 10 mL each were collected; compound **2** (10.7 mg) was obtained after purification of sub-fr 64-70 on sephadex LH20. Similar treatment of TTFb_4_ (1.56 g) gave compounds **8**(55.4 mg; sub-frs 100-110), **6**(32.6 mg; sub-frs 170-184), **7** (5.3 mg; sub-frs 185-189) and **9** (6.8 mg; sub-frs 190-200) which were purified on sephadex LH20.

***S2. Physical properties and NMR data of compounds 1 – 9.***

Compound (**1**): (3*R*, 4*S*)-3,4-dimethyloxetan-2-one (C_5_H_8_O_2_); yellow paste soluble in MeOH, HRESMS [M+H]^+^ at *m/z* : 101.0598. ^1^H NMR (400 MHz, MeOD), *δ* (ppm): 3.94 (1H, quint, *J* = 6.4, 0.8 Hz, H-4), 2.45 (1H, quint, *J* = 7.2, 6.8 Hz, H-3), 1.19 (3H, d, *J* = 6.4 Hz, CH_3_), 1.13 (3H, d, *J* = 6.8, CH_3_). ^13^C NMR (400 MHz, MeOD) , *δ* (ppm): 177.5 (C-2), 68.5 (C-4), 47.0 (C-3), 18.7 (**C**H_3_-C-4), 11.9 (**C**H_3_-C-3) [1]


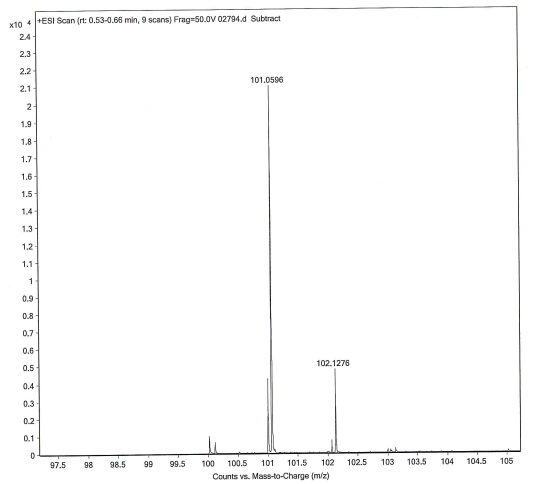


Figure S1: HRESIMS of compound **1**.

Figure S2: ^1^H NMR spectrum (400 MHz, MeOD) of compound **1**.

Figure S3: ^13^C NMR spectrum (100 MHz, DMSO) of compound **1**.

Figure S4: COSY spectrum of compound **1**.

Figure S5: HSQC spectrum of compound **1**.

Figure S6: HMBC spectrum of compound **1**.

Compound **(2**): Luteolin or 5,7,3′,4′-tetrahydroxyflavone (C_15_H_10_O_6_); yellow powder soluble in MeOH, ESI-MS [M+H] peak at *m/z* 287.1. mp: 330-332 ^o^C. PubChem CID: 5280445.

^1^H NMR (600 MHz, DMSO), *δ* (ppm): 7.34 (1H, d, *J* = 2.4 Hz, H-2′), 7.32 (1H, dd, *J* = 8.4, 2.4 Hz, H-6′), 6.85 (1H, d, *J* = 8.4 Hz, H-5′), 6.54 (1H, s, H-3), 6.39 (1H, d, *J* = 2.4 Hz, H-8), 6.14 (1H, d, *J* = 2.4 Hz, H-6). ^13^C NMR (150 MHz, DMSO), (ppm): 182.0 (C-4), 164.3 (C-7), 164.3 (C-2), 161.6 (C-5), 157.7 (C-9), 149.5 (C-4′), 145.6 (C-3′), 122.1 (C-1′), 119.0 (C-6′), 115.8 (C-5′), 113.0 (C-2′), 103.9 (C-10), 102.9 (C-3), 98.7 (C-6), 93.8 (C-8) [2]

.


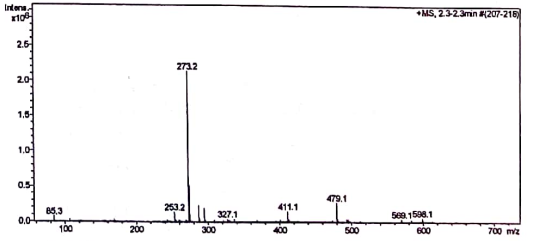


Figure S7: ESIMS spectrum of compound **2**.

Figure S8: ^1^H NMR spectrum (600 MHz, DMSO) of compound **2**.

Figure S9: ^13^C NMR spectrum (150 MHz, DMSO) of compound **2**.

Compound (**3**): Stigmasterolor stigmasta-5,22(*E*)-dien-3*α*-ol (C_29_H_48_O); white solid soluble in CDCl_3_,^1^H and ^13^C NMR data: ESI-MS [M+H] peak at *m/z* 413.8. mp: 170-171^o^C. PubChem CID: 5280794

^1^H NMR (CDCl_3_, 600 MHz), *δ* (ppm): 5.37 (1H, q, *J* = 3.6, 2.4 Hz, H-6), 5.16 (1H, q, *J* = 9.0, 3.0 Hz, H-22), 5.04 (1H, q, *J* = 9.0, 6.0 Hz, H-23), 3.55 (1H, m, H-3), 1.04 (3H, t, H-27), 1.03 (3H, d, *J* = 6.6 Hz, H-21), 0.94 (3H, d, H-28), 0.86 (3H, d, *J* = 6.5 Hz, H-26), 0.82 (3H, t, *J* = 6.0 Hz, H-29), 0.82 (3H, d, *J* = 6.0 Hz, H-19), 0.71 (3H, s, H-18). ^13^C NMR (CDCl_3_, 150 MHz), *δ* (ppm):140.7 (C-5), 138.3 (C-22), 129.2 (C-23), 121.7 (C-6), 71.8 (C-3), 56.8 (C-14), 55.9 (C-17), 51.3 (C-24), 50.1 (C-9), 42.2 (C-4), 42.2 (C-13), 40.5 (C-20), 39.6 (C-12), 37.1 (C-1), 36.5 (C-10), 31.9 (C-25), 31.8 (C-7), 31.6 (C-8), 29.7 (C-2), 28.9 (C-16), 25.4 (C-28), 24.3 (C-15), 21.2 (C-19), 21.1 (C-11), 21.0 (C-26), 19.4 (C-27), 18.9 (C-28), 12.2 (C-29), 12.0 (C-18) [3].

.

Figure S10: ^1^H NMR spectrum (600 MHz, CDCl_3_) of compound **3**.

Figure S11: ^13^C NMR spectrum (150 MHz, CDCl_3_) of compound **3**.

Compound (**4**): 3-*O*-[6’-*O*-undecanoyl-*β*-_D_-glucopyranosyl]stigmasterol (C_46_H_78_O_7_), tansparent crystals soluble in chloroform. HRESMS [M-H +Na]^+^ at *m/z*: 764.5682. ^1^H NMR (CDCl_3_, 600 MHz), *δ* (ppm): 5.40 (1H, d, *J* = 9.6 Hz, H-6), 5.19 (1H, dd, *J* = 16.2, 10.8, H-22), 5.08 (1H, dd, *J* = 13.2, 4.2 Hz, H-23), 3.49 (1H, m, H-3), 1.04 (3H, t, H-27), 1.03 (3H, d, *J* = 6.6 Hz, H-21), 0.94 (3H, d, H-28), 0.86 (3H, d, *J* = 6.5 Hz, H-26), 0.82 (3H, t, *J* = 6.0 Hz, H-29), 0.82 (3H, d, *J* = 6.0 Hz, H-19), 0.71 (3H, s, H-18).* β-*Gluc: 4.43 (1H, d, *J* = 6.0 Hz, H-6′), 4.40 (1H, d, *J* = 10.8 Hz, H-1′) 4.35 (1H, d, H-6′b), 3.59 (1H, m, H-5′), 3.49 (1H, t, H-4′), 3.40 (1H, t, *J* = 16.8 Hz, H-2′). ^13^C NMR (CDCl_3_, 150 MHz) *δ* (ppm): 140.3 (C-5), 138.3 (C-22), 129.3 (C-23), 122.1 (C-6), 73.4 (C-3), 56.8 (C-14), 55.9 (C-17), 51.2 (C-24), 50.1 (C-9), 42.2 (C-4), 42.2 (C-13), 40.5 (C-20), 38.9 (C-12), 37.2 (C-1), 36.7 (C-10), 31.9 (C-25), 31.8 (C-7), 31.6 (C-8), 29.7 (C-2), 28.9 (C-16), 25.4 (C-28), 25.0 (C-15), 21.2 (C-19), 21.1 (C-11), 21.0 (C-26), 19.3 (C-27), 19.0 (C-28), 12.2 (C-29), 12.0 (C-18).* β-*Gluc: 101.2 (C-1′), 79.6 (C-5′), 76.0 (C-2′), 73.8 (C-3′), 70.1 (C-4′), 63.3 (C-6′). Saturated fatty acid 174.5 (C-1″), 34.2 (C-2″), 32.0, 29.4-29.2 [(CH_2_)n], 14.1 (CH_3_- term) [4].

Figure S12: ^1^H NMR (300 MHz, CDCl_3_) spectrum of compound **4**.

Figure S13: ^13^C NMR (75 MHz, CDCl_3_) spectrum of compound **4**

Figure S14: ^13^C APT NMR spectrum (75 MHz, CDCl_3_) of compound **4**.

Compound (**5**): olean-12-en-3-*β*-*O*-_D_-glucopyranoside (C_36_H_60_O_6_); white powder soluble in MeOH. ^1^H NMR (MeOD, 400 MHz) *δ* (ppm): 5.24 (1H, t, *J* = 3.6 Hz, H-12), 3.17 (1H, m, H-3), 2.84 (1H, dd, H-18), 1.89 (1H, dd, H-11a), 1.62 (1H, dd, H-11b), 1.17 (3H, s, H-27), 1.07 (3H, s, H-24), 0.97 (3H, s, H-19), 0.96 (3H, s, H-29), 0.92 (3H, s, H-30), 0.86 (3H, s, H-23), 0.84 (3H, s, H-28), 0.82 (3H, s, H-26), 0.79 (1H, *br*s, H-5). *β-*Gluc: 4.32 (1H, dd, *J* = 7.6, 2.0 Hz, H-1′) 3.83 (1H, dd, H-6′a), 3.66 (1H, dd, H-6′b), 3.32 (1H, m, H-5′), 3.27 (1H, t, H-4′), 3.25 (1H, t, H-2′). ^13^C NMR (MeOD, 100 MHz) *δ* (ppm): 143.8 (C-13), 122.2 (C-12), 89.3 (C-3), 55.6 (C-5), 46.9 (C-9), 46.2 (C-17), 45.8 (C-19), 41.4 (C-14), 41.3 (C-18), 39.1 (C-8), 38.7 (C-4), 38.3 (C-1), 36.4 (C-10), 33.5 (C-21), 32.6 (C-7), 32.4 (C-22), 32.2 (C-29), 30.2 (C-20), 27.4 (C-15), 27.1 (C-24), 25.6 (C-16), 24.9 (C-27), 23.1 (C-11), 22.6 (C-30), 17.9 (C-6), 16.3 (C-26), 15.9 (C-28), 15.5 (C-23), 14.5 (C-25). *β-*Gluc: 105.3 (C-1′), 76.8 (C-2′), 76.2 (C-5′), 74.2 (C-3′), 70.2 (C-4′), 61.3 (C-6′) [5].

Figure S15: ^1^H NMR spectrum (400 MHz, MeOD) of compound **5**

Figure S16: ^13^C NMR spectrum (100 MHz, MeOD) of compound **5**.

Compound (**6**): 3-O-*β*-_D_-glucopyranosyl-(1→6)-*β*-_D_-glucopyranosylurs-12-en-28-oic acid (C_42_H_68_O_13_); white powder soluble in MeOH, HRESMS [M+Na]^+^ at m/z: 803.4478.

^1^H NMR (MeOD, 400 MHz) *δ* (ppm): 5.65 (1H, t, *J* = 2.8 Hz, H-12), 3.25 (1H, m, H-3), 2.91 (1H, dd, H-18), 1.90 (1H, dd, H-11a), 1.54 (1H, dd, H-11b), 1.18 (3H, s, H-27), 1.07 (3H, s, H-24), 0.95 (3H, d, H-30), 0.93 (3H, s, H-25), 0.91 (3H, d, H-29), 0.86 (3H, s, H-23), 0.79 (3H, s, H-26), 0.89 (1H, *br*s, H-5). *β-*Gluc I: 4.36 (1H, d, *J* = 8.0 Hz, H-1′) 4.09 (1H, dd, *J* = 12.0, 2.0 Hz, H-6′a), 3.80 (1H, dd, *J* = 12.0, 2.0 Hz, H-6′b), 3.46 (1H, t, H-3′), 3.35 (1H, m, H-5′), 3.29 (1H, t, H-4′), 3.19 (1H, t, H-2′).* β-*Gluc II: 4.48 (1H, dd, *J* = 12.0, 2.0 Hz, H-1′) 3.86 (1H, dd, *J* = 12.0, 2.0 Hz, H-6′a), 3.66 (1H, dd, *J* = 5.6, 2.0 Hz, H-6′b), 3.45 (1H, m, H-3′), 3.34 (1H, m, H-5′), 3.37 (1H, t, H-2′), 3.28 (1H, t, H-4′). ^13^C NMR (MeOD, 100 MHz) *δ* (ppm): 180.2 (C-28), 138.1 (C-13), 127.9 (C-12), 88.4 (C-3), 55.2 (C-5), 46.9 (C-9), 45.9 (C-17), 44.9 (C-19), 41.5 (C-18), 40.9 (C-14), 39.1 (C-8), 38.7 (C-4), 38.3 (C-1), 36.6 (C-10), 33.5 (C-22), 32.8 (C-7), 32.4 (C-21), 32.1 (C-29), 30.2 (C-20), 27.4 (C-15), 27.0 (C-24), 25.7 (C-16), 25.0 (C-27), 23.4 (C-11), 22.6 (C-30), 17.7 (C-6), 17.4 (C-26), 15.6 (C-23), 14.8 (C-25). *β-*Gluc I: 105.2 (C-1′), 76.4 (C-2′), 76.7 (C-5′), 74.2 (C-3′), 70.1 (C-4′), 68.3 (C-6′); *β-*Gluc II: 103.5 (C-1′), 76.5 (C-2′), 75.9 (C-5′), 73.6 (C-3′), 70.2 (C-4′), 61.4 (C-6′) [6].

Figure S17: ^1^H NMR spectrum (400 MHz, MeOD) of compound **6**.

Figure S18: ^13^C NMR spectrum (100 MHz, MeOD) of compound **6**.

Compound (**7**): 3-*O*-*β*-_D_-glucopyranosyl-(1→3)-*β*-_D_-glucopyranosyl-27-hydroxyolean-12-en-28-oic acid ^1^H NMR (MeOD, 400 MHz) *δ* (ppm): 5.24 (1H, *br*s , H-12), 3.76 (1H, d, H-27a), 3.44 (1H, d, H-27b), 3.03 (1H, m, H-3), 2.87 (1H, dd, H-18), 1.89, 1.62 (2H, dd, H-11), 1.17 (3H, s, H-27), 0.97 (3H, s, H-24), 0.95 (3H, s, H-30), 0.94 (3H, s, H-25), 0.91 (3H, d, H-29), 0.89 (3H, s, H-26), 0.77 (3H, s, H-23), 0.76 (1H, s, H-5). *β-*Gluc I: 4.29 (1H, d, *J* = 7.6 Hz, H-1′) 3.81 (1H, d, *J* = 11.6 Hz, H-6′a), 3.67 (1H, d, *J* = 11.6 Hz, H-6′b), 3.57 (1H, m, H-5′), 3.54 (1H, t, H-2′) 3.46 (1H, t, H-3′), 3.25 (1H, t, H-4′).* β-*Gluc II: 4.43 (1H, d, *J* = 8.0 Hz, H-1′), 4.41 (1H, d, *J* = 12.0 Hz, H-6′a), 3.66 (1H, d, *J* = 12.0 Hz, H-6′b), 3.47 (1H, m, H-3′), 3.45 (1H, m, H-5′), 3.37 (1H, t, H-2′), 3.28 (1H, t, H-4′).^13^C NMR (MeOD, 100 MHz): 180.4 (C-28), 145.2 (C-13), 122.0 (C-12), 89.8 (C-3), 63.4 (C-27), 55.6 (C-5), 47.7 (C-9), 46.2 (C-17), 45.7 (C-19), 41.4 (C-14), 41.3 (C-18), 39.1 (C-8), 38.5 (C-4), 38.4 (C-1), 36.4 (C-10), 33.4 (C-22), 32.4 (C-21), 32.1 (C-7), 32.1 (C-29), 30.1 (C-20), 29.2 (C-2), 27.4 (C-15), 27.1 (C-24), 23.1 (C-11), 22.5 (C-30), 21.6 (C-16), 17.9 (C-6), 11.3 (C-26), 15.5 (C-23), 14.4 (C-25). *β-*Gluc I: 103.9 (C-1′), 80.1 (C-3′), 75.7 (C-5′), 73.2 (C-2′), 71.1 (C-4′), 68.9 (C-6′); *β-*Gluc II: 103.4 (C-1′), 76.5 (C-2′), 75.8 (C-5′), 73.9 (C-3′), 71.0 (C-4′), 61.1 (C-6′) [7].

Figure S19: ^1^H NMR spectrum (400 MHz, MeOD) of compound **7**.

Figure S20: ^13^C NMR spectrum (100 MHz, MeOD) of compound **7**.

Figure S21: HSQC spectrum of compound **7**.

Compound (**8**): * *methyl-O-*β*-_D_-glucopyranoside, (C_7_H_14_O_6_); white crystals soluble in MeOH, HRESMS [M+Na]^+^ at *m/z* 217.0720 calcd 194.18. PubChem CID: 3036743. ^1^H NMR (DMSO, 400 MHz) *δ* (ppm): 4.01 (1H, d, *J* = 7.6 Hz, H-1), 3.64 (1H, dd, *J* = 6.0, 2.0 Hz, H-6a), 3.42 (1H, dd, *J* = 6.0, 2.0 Hz, H-6b), 3.36 (3H, s, H-1′/CH_3_-O), 3.18 (1H, m, H-5), 3.05 (1H, m, H-2), 3.02 (1H, m, H-4), 2.91 (1H, m, H-3). ^13^C NMR (MeOD, 100 MHz) *δ* (ppm): 104.4 (C-1), 77.2 (C-5), 77.0 (C-2), 73.8 (C-3), 70.4 (C-4), 61.5 (C-6), 56.4 (C-1′) [8].

Figure S22: ^1^H NMR spectrum (400 MHz, MeOD) of compound **8**.

Figure S23: ^13^C NMR spectrum (100 MHz, MeOD) of compound **8**.

Compound (**9**): *β*-_D_-fructofuranosyl-(2→1)-*β*-_D_-glucopyranoside (C_12_H_22_O_10_); white crystals, soluble in MeOH. ^1^H NMR (MeOD, 400 MHz) *δ* (ppm): 5.41 (1H, d, *J* = 4.0 Hz, H-1), 3.41 (1H, m H-2), 3.83 (1H, m, H-3), 3.35 (1H, m, H-4), 3.70 (1H, m, H-5), 3.76 (2H, m, H-6); *β*-fructofuranosyl: 3.61 (1H, d, *J* = 5.6 Hz, H-1′), 4.11 (1H, d, H-3′), 3.78 (1H, dd, H-4′), 3.76 (1H, m, H-5′), 3.79 (1H, m, H-6′). ^13^C NMR (MeOD, 100 MHz) *δ* (ppm): 103.9 (C-2′), 92.2 (C-1), 82.3 (C-5′), 77.8 (C-3′), 74.2 (C-4′), 73.2 (C-5), 72.9 (C-3), 71.8 (C-2), 69.8 (C-4), 62.6 (C-1′), 61.9 (C-6), 60.7 (C-6′) [9].

Figure S24: ^1^H NMR spectrum (400 MHz, MeOD) of compound **9**.

Figure S25: ^13^C NMR spectrum (100 MHz, MeOD) of compound **9**.


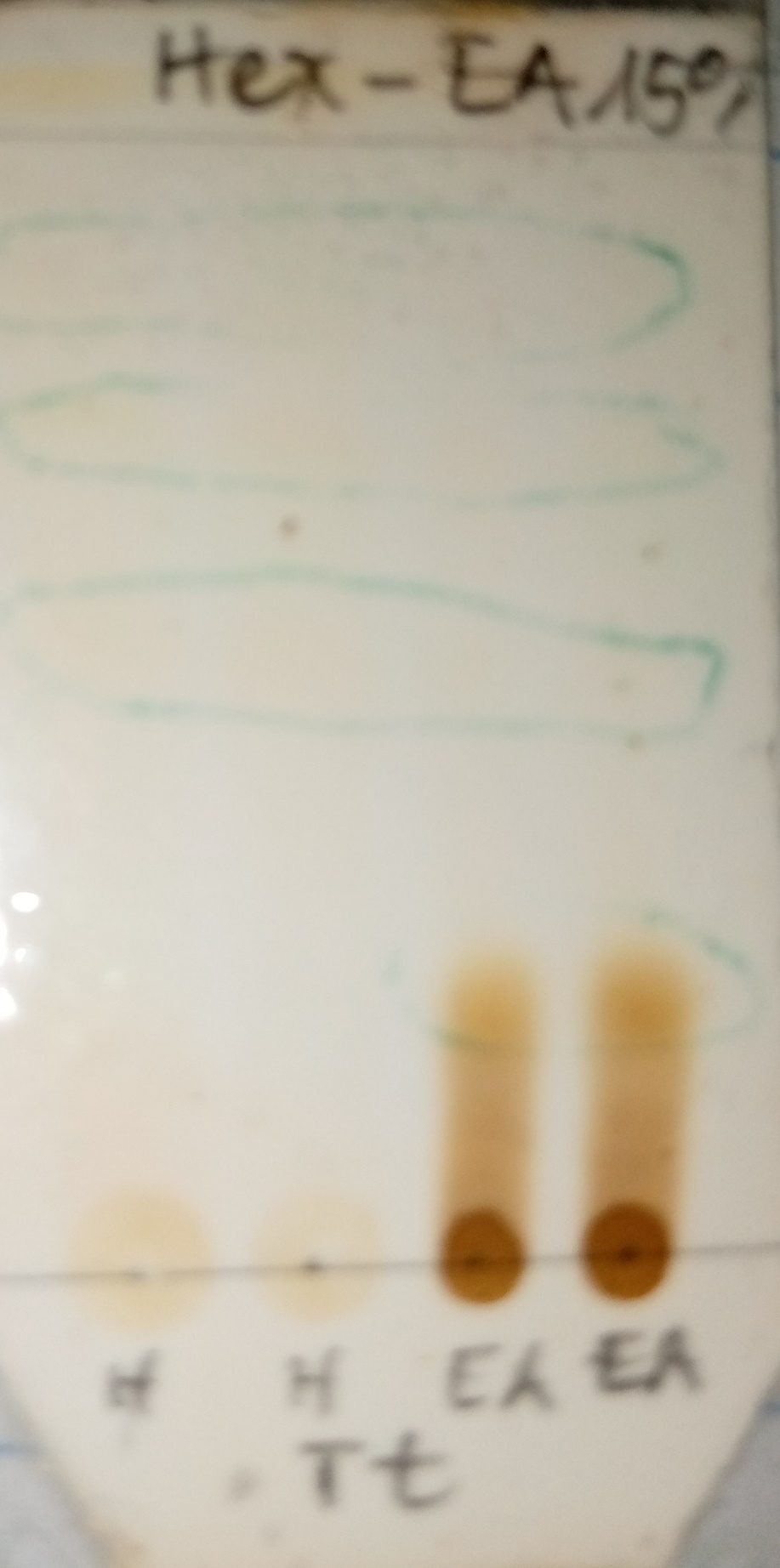


Figure S26: TLC profiles for Hexane and Ethyl Acetate extracts.


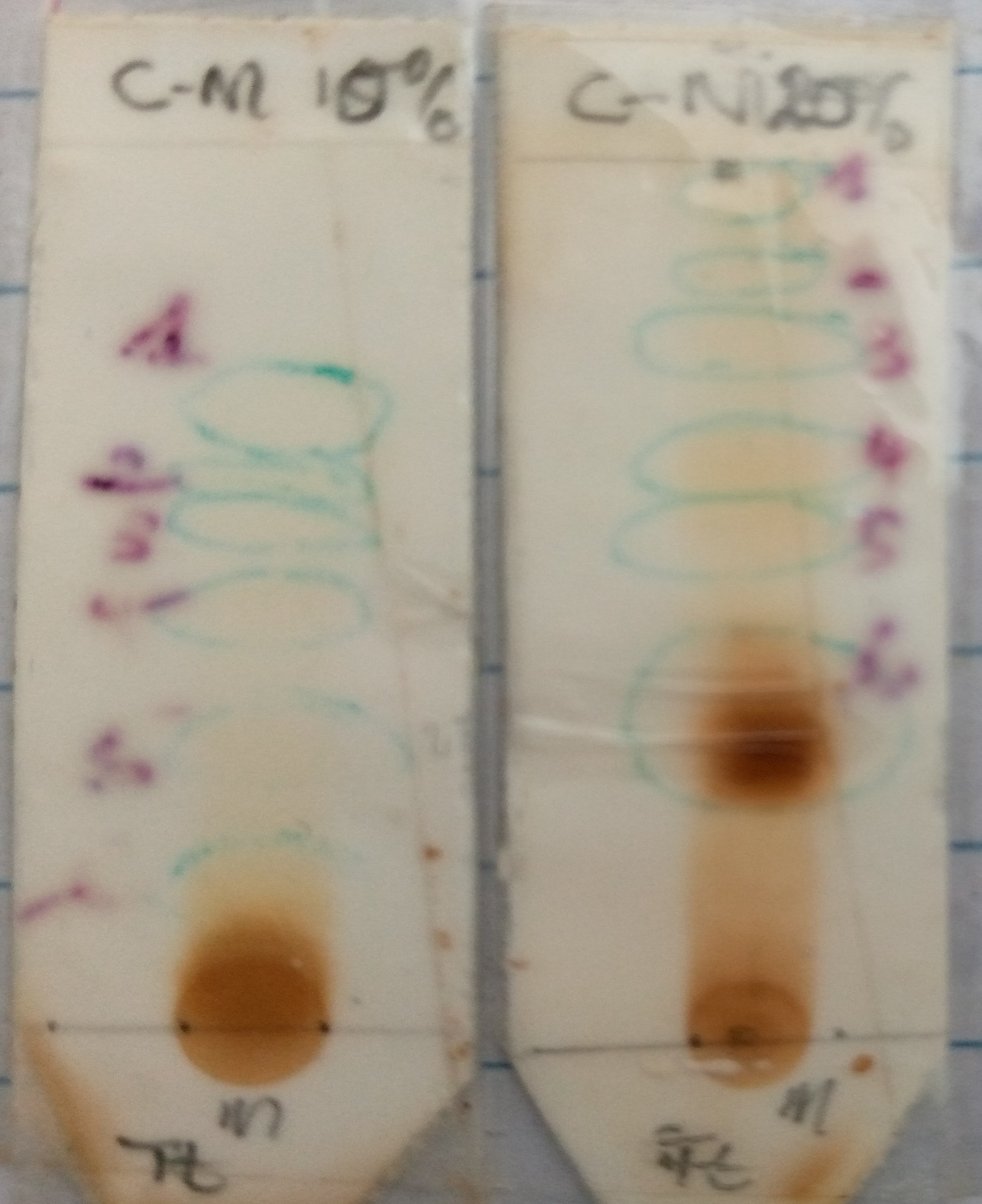


Figure S27: TLC profiles for Methanol extract.

**References**

1. Case-Green SC, Davies SG, Roberts PM, Russell AJ, Thomson JE. Asymmetric synthesis of tetrahydrolipstatin and valilactone. Tetrahedron: Asymmetry. 2008; 19, 2620-2631.
2. Antonio L, Gabriela L, Henrique F, Conceic AD, Barison A, Weber M. Phytochemical analysis of Vernonanthura tweedieana and a validated UPLC-PDA method for the quantification of eriodictyol. Rev Bras Farm. 2015; 25, 375–381.
3. Pierre LL, Moses MN. Isolation and Characterisation of Stiglasterol and B-Sitosterol from odontonema Strictum (Acanthaceae). J Innov Pharm Biol Sci. 2015; 2, 88-95.
4. Gomes DDCF, Alegrio LV. Acyl steryl glycosides from *Pithecellobium cauliflorum*. Phytochemistry. 1998; 49, 1365-1367.
5. Afzal M, Gupta G, Kazmi I, Rahman M, Afzal O, Alam J, Hakeem KR, Pravez M, Gupta R, Anwar F. Anti-inflammatory and analgesic potential of a novel steroidal derivative from *Bryophyllum pinnatum*. Fitoterapia. 2012; 83, 853-858.
6. Lunga PK, Qin XJ, Yang XW, Kuiate JR, Du ZZ, Gatsing D. A New antimicrobial and radical-scavenging glycoside from *Paullinia pinnata* var. cameroonrnsis. Nat Prod Res. 2015; 29, 1688-1694.
7. Aladesanmi AJ. *Tetrapleura tetraptera:* molluscidal activity and chemical constituents. Afr J Tradit Complement Altern Med. 2006; 4, 23-26.
8. Aubert S, Choler P, Pratt J, Douzet E, Bligny R. Methyl-beta-D-glucopyranoside in higher plants: accumulation and intracellular localization in Geummontanum L. leaves and in model systems studied by ^13^C nuclear magnetic resonance. J Exp Bot. 2004; 55, 2179-2189.

9. Jones AJ, Hanisch P, McPhail AK. Sucrose: An assignment of the ^13^C N.M.R. parameters by selective decoupling. Austrian J Chem. 1979; 32, 2763-2764.
