# Supplementary material for: Cytotoxicity, acute and sub-chronic toxicities of the fruit extract of Tetrapleura tetraptera (Schumm. & Thonn.) Taub. (Fabaceae)
Source: BMC Complement Med Ther. 2022 Jul 4;22:178. doi: 10.1186/s12906-022-03659-1 (PMC9252075; doi:10.1186/s12906-022-03659-1)

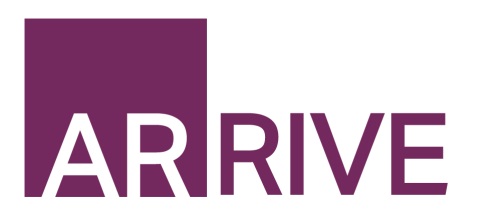


The ARRIVE Guidelines Checklist

Animal Research: Reporting In Vivo Experiments

Carol Kilkenny^1^, William J Browne^2^, Innes C Cuthill^3^, Michael Emerson^4^ and Douglas G Altman^5^

*^1^The National Centre for the Replacement, Refinement and Reduction of Animals in Research, London, UK, ^2^School of Veterinary Science, University of Bristol, Bristol, UK, ^3^School of Biological Sciences, University of Bristol, Bristol, UK, ^4^National Heart and Lung Institute, Imperial College London, UK, ^5^Centre for Statistics in Medicine, University of Oxford, Oxford, UK.*

|  | ITEM | RECOMMENDATION | Section/ Paragraph | |  |
| --- | --- | --- | --- | --- | --- |
| 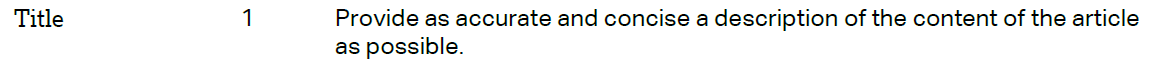 | | | | 1. Title | |
| 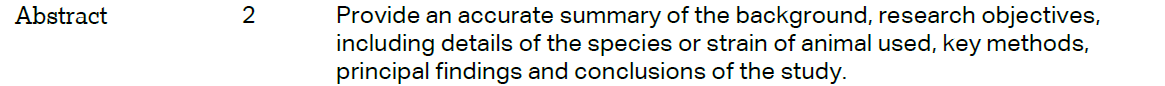 | | | | 2. Abstract | |
| INTRODUCTION | | | |  | |
| 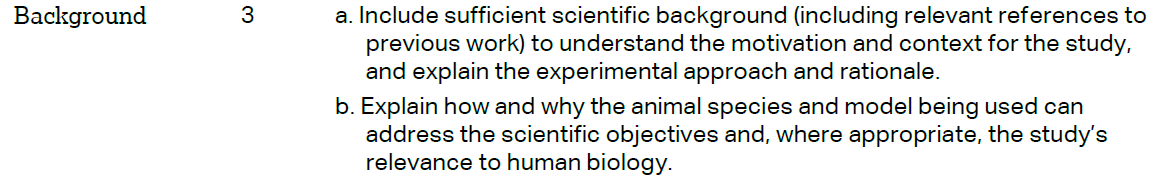 | | | | 3a. Background, Paragraph 1  3b Background, Paragraph 2 | |
| 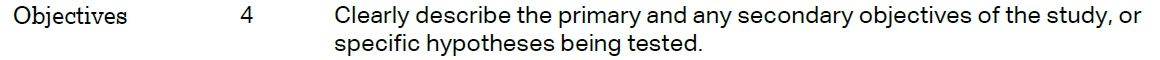 | | | | 4. Objective, Last paragraph | |
| METHODS | | | |  | |
| 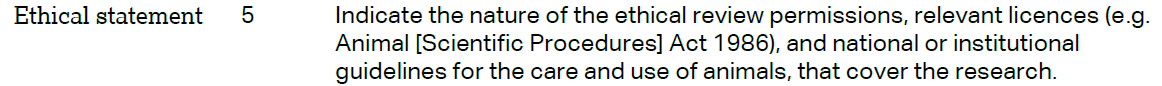 | | | | 5. Methods section, experimental animals’ subsection | |
| 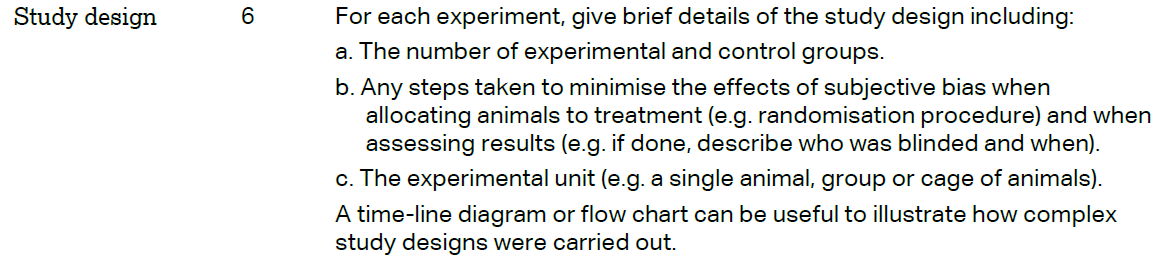 | | | | 6a. Methods section, acute oral toxicity study subsection, study of sub-chronic oral toxicity subsection  6b. Methods section   - Acute oral toxicity study subsection - Study of sub-chronic oral toxicity subsection - Hematological parameters subsection - Biochemical parameters subsection - Histological analysis subsection   6c. Methods section   - Acute oral toxicity study subsection (5 females’ rats for the test group and 5 females’ rats for the control group) - Study of sub-chronic oral toxicity subsection (4 groups of 8 animals per group: 4 females and 4 males). | |
| 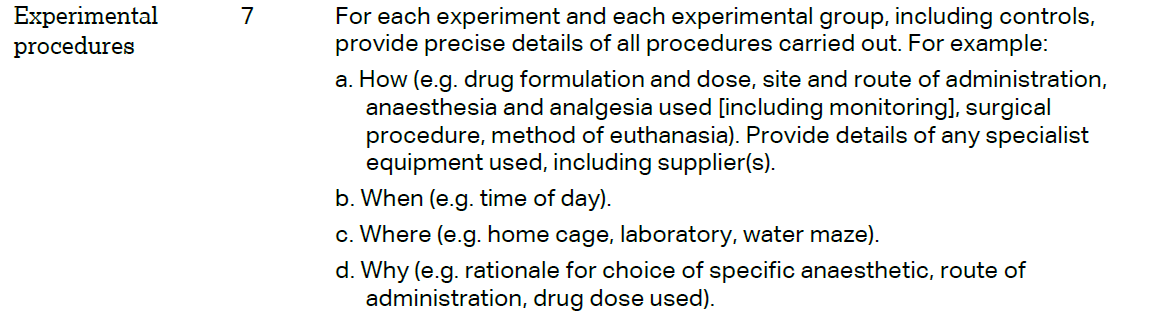 | | | | 7a. Methods section   - Acute oral toxicity study subsection - Study of sub-chronic oral toxicity subsection   7b. Methods section   - Acute oral toxicity study subsection - Study of sub-chronic oral toxicity subsection   7c. Methods section, experimental animals’ subsection  7d. Methods section   - Acute oral toxicity study subsection - Study of sub-chronic oral toxicity subsection | |
| 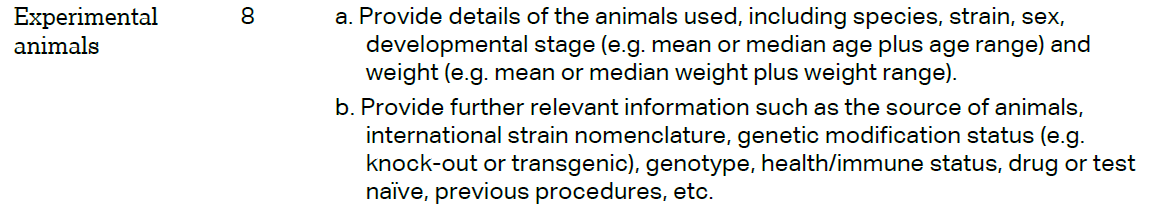 | | | | 8a. Methods section   - Acute oral toxicity study subsection - Study of sub-chronic oral toxicity subsection   8b. Methods section, Acute oral toxicity study subsection (animals) | |

The ARRIVE guidelines. Originally published in *PLoS Biology*, June 2010^1^

| 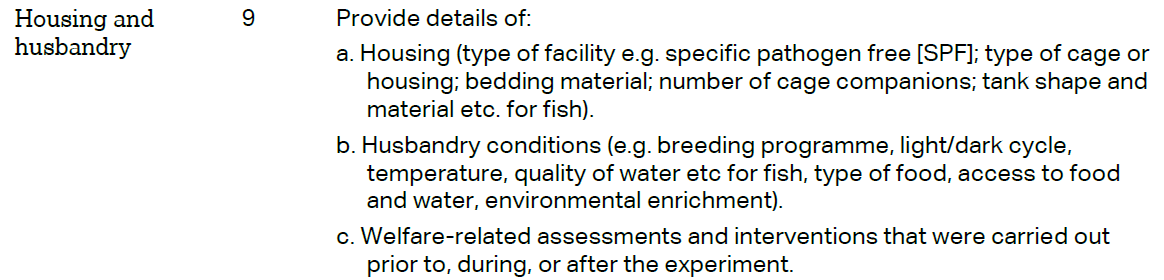 | 9a. Methods section   - Acute oral toxicity study subsection - Sub-chronic oral toxicity study subsection   9b. Methods section, experimental animals’ subsection  9c. Methods section: Acute oral toxicity study subsection and Study of sub-chronic oral toxicity subsection | |
| --- | --- | --- |
| 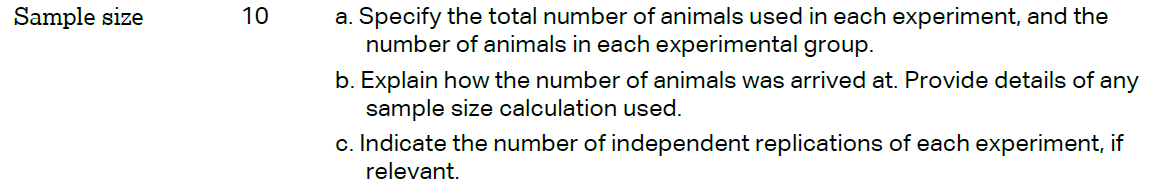 | 10a. Method section   - Methods section: Acute oral toxicity study subsection and Study of sub-chronic oral toxicity subsection   10b. Methods section  10c. Methods section   - Acute oral toxicity study subsection - Study of sub-chronic oral toxicity   subsection | |
| 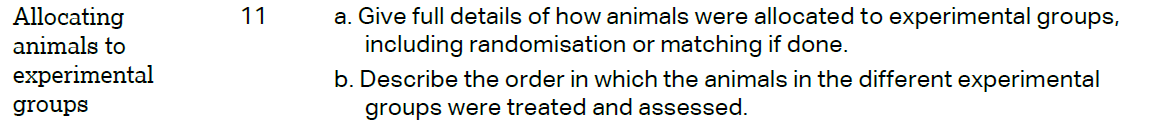 | 11a. Methods section   - Experimental animals - Acute oral toxicity study subsection - Study of sub-chronic oral toxicity subsection   11b. Methods section   - Acute oral toxicity study subsection - Study of sub-chronic oral toxicity subsection | |
| 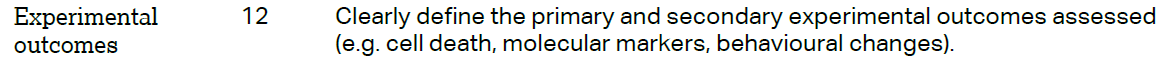 | 12. Methods section   - Acute oral toxicity study subsection - Study of sub-chronic oral toxicity subsection - Assessment of Hematological parameters subsection - Assessment of Biochemical parameters subsection - Histological analysis subsection | |
| 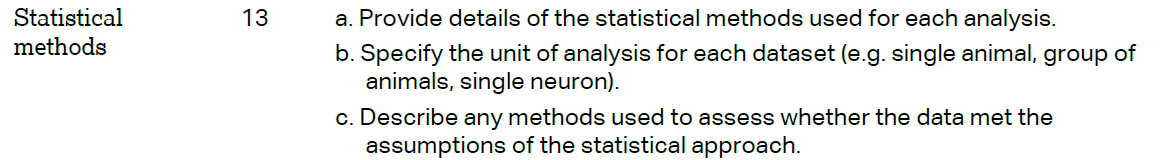 | 13a. Methods section: statistical analysis subsection  13b. Methods section: statistical analysis subsection  13c. Methods section: statistical analysis subsection | |
| RESULTS |  | |
| 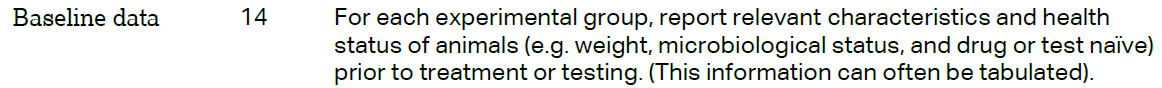 | 14. Results section, Tables 2-6 (dose in mg/kg); Figures 2 and 3 | |
| 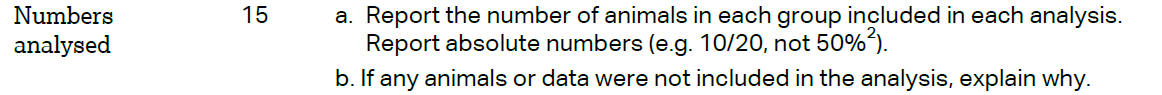 | 15a. Throughout results Section  15b. Not applicable | |
| 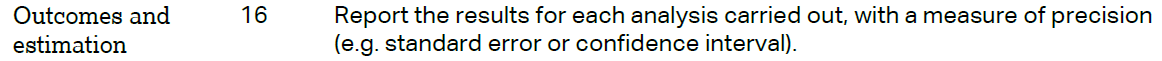 | 16. Results section, Tables 2-6; Figure 2-6 | |
| 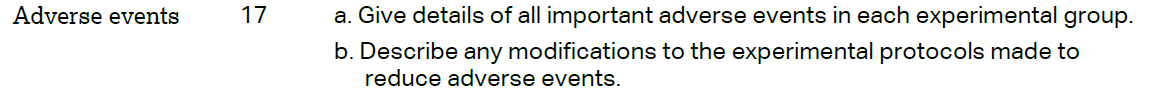 | 17. Not applicable | |
| DISCUSSION |  | |
| 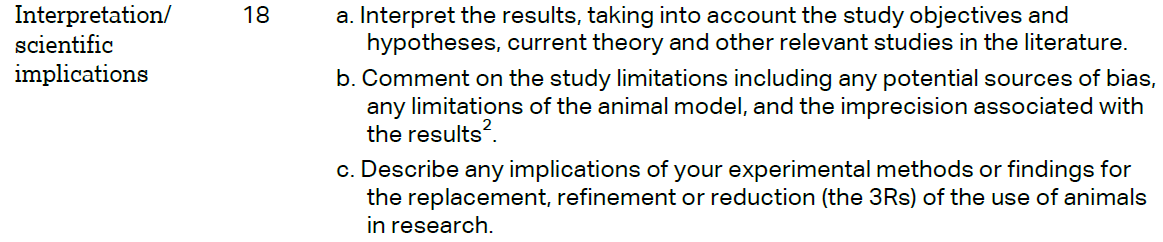 | 18. Throughout Discussion Section | |
| 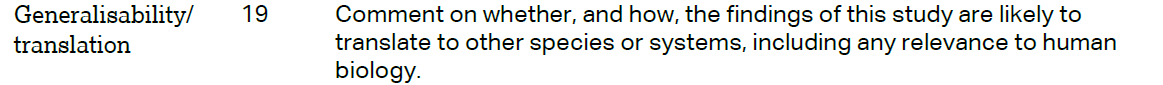 | 19. Not applicable | |
| 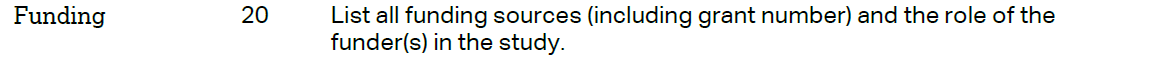 | | 20. No funding |


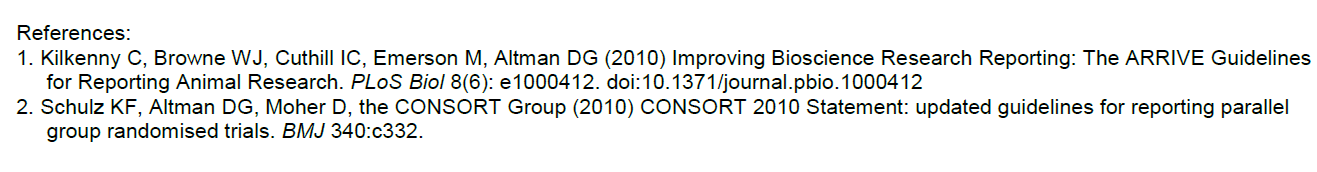

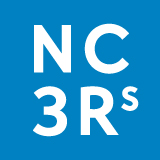

Supplement: Supplementary file 2 — Additional file 2. [file 12906_2022_3659_MOESM2_ESM.docx]
